# Noxa and Mcl-1 expression influence the sensitivity to BH3-mimetics that target Bcl-xL in patient-derived glioma stem cells

**DOI:** 10.1038/s41598-022-20910-4

**Published:** 2022-10-22

**Authors:** Mariana Belén Vera, Olivia Morris-Hanon, Germán Ignacio Nogueiras, Luisina Belén Ripari, Myrian Inés Esquivel, Carolina Perez-Castro, Leonardo Romorini, Gustavo Emilio Sevlever, María Elida Scassa, Guillermo Agustín Videla-Richardson

**Affiliations:** 1 Laboratorio de Investigación aplicada a Neurociencias (LIAN), Fundación para la Lucha contra las Enfermedades Neurológicas de la Infancia (FLENI), Ruta 9, Km 52.5, B1625XAF Buenos Aires, Argentina; 2 Instituto de Investigación en Biomedicina de Buenos Aires (IBioBA)-CONICET-Partner Institute of the Max Planck Society, Godoy Cruz 2390, C1425FQD Ciudad de Buenos Aires, Argentina

**Keywords:** Cancer stem cells, Tumour heterogeneity, Apoptosis

## Abstract

The recurrence of Glioblastoma is partly attributed to the highly resistant subpopulation of glioma stem cells. A novel therapeutic approach focuses on restoring apoptotic programs in these cancer stem cells, as they are often deregulated. BH3-mimetics, targeting anti-apoptotic Bcl-2 family members, are emerging as promising compounds to sensitize cancer cells to antineoplastic treatments. Herein, we determined that the most abundantly expressed anti-apoptotic Bcl-2 family members, Bcl-xL and Mcl-1, are the most relevant in regulating patient-derived glioma stem cell survival. We exposed these cells to routinely used chemotherapeutic drugs and BH3-mimetics (ABT-263, WEHI-539, and S63845). We observed that the combination of BH3-mimetics targeting Bcl-xL with chemotherapeutic agents caused a marked increase in cell death and that this sensitivity to Bcl-xL inhibition correlated with Noxa expression levels. Interestingly, whereas co-targeting Bcl-xL and Mcl-1 led to massive cell death in all tested cell lines, down-regulation of Noxa promoted cell survival only in cell lines expressing higher levels of this BH3-only. Therefore, in glioma stem cells, the efficacy of Bcl-xL inhibition is closely associated with Mcl-1 activity and Noxa expression. Hence, a potentially effective strategy would consist of combining Bcl-xL inhibitors with chemotherapeutic agents capable of inducing Noxa, taking advantage of this pro-apoptotic factor.

## Introduction

Glioblastoma multiforme (GBM), a Grade IV-Diffuse Glioma, is an aggressive primary brain tumor^1,2^. Even with great advances in surgical procedures and treatments, high-grade gliomas remain incurable. The standard of care protocol for newly diagnosed GBM is surgical resection followed by radio- and chemotherapy, in which temozolomide (TMZ) is the main chemotherapeutic drug^3^. In addition, a multi-drug chemotherapy composed by procarbazine, lomustine (CCNU) and vincristine (VCR), is commonly used in recurrent high-grade gliomas^4^.

GBM is characterized by an abundant inter- and intra-tumor heterogeneity. GBM comprises subpopulations of cancer cells with distinct proliferation rates, differentiation, and tumorigenic potential^5^. Among them, glioma stem cells (GSC) are highly tumorigenic cells characterized by their ability to self-renew and their capacity to differentiate into a wide variety of cells^6,7^. This multipotency partially contributes to the intra-tumor heterogeneity observed in GBM^5^. Importantly, GSCs also exhibit an increased radio- and chemoresistance^8^. Therefore, to avoid GBM recurrence, it is critical to target biological processes that sustain GCS viability.

Previously, we established several patient-derived glioma stem cell-enriched cell lines (GSE-ECL) and determined that these cultures exhibit several features of cancer stem cells; including self-renewal, neurosphere formation, expression of stem cell markers, differentiation potential and tumorigenic capacity^9^. We also described that these cell lines show differential sensitivities to different pro-apoptotic stimuli^10^.

Bcl-2 (B-cell lymphoma 2) family members regulate apoptotic programs^11^. These proteins are grouped into three classes based on their functions and sequence homology: the pro-survival Bcl-2-like proteins (Bcl-2, Bcl-xL, Mcl-1, Bfl-1, and Bcl-w), the pro-apoptotic multi-domain factors (Bax, Bak, and Bok), and the single domain BH3-only proteins (Bim, Puma, Bid, Bad, Bik, Bmf, Noxa, and Hrk)^11,12^. Molecules that target apoptotic pathways have been proven to have an anticancer effect in a wide range of malignancies, many of which are now entering clinical trials. As such, increasing attention has focused on developing therapeutic strategies that specifically target the apoptotic pathway in GBM^13,14^.

BH3-only factors relay upstream apoptotic signals to initiate apoptosis, either by activating Bax and Bak directly, such as Bim and Bid (activator BH3-only proteins), or by inactivating pro-survival Bcl-2-like factors, such as Bad, Bmf, Bik, Hrk, Noxa and Puma (sensitizer BH3-only proteins)^15,16^. In addition, some BH3-only proteins, such as Puma, Bmf, and Noxa, have also been described as direct activators of Bak/Bax^17–20^. However, these findings are somewhat controversial since some reports propose that these BH3-only factors, as well as Bid and Bim, are only sensitizers^21,22^. Therefore, the complexity of the Bcl-2 family and the difficulty in evaluating its physiological relevance hinder the elucidation of the mechanisms of action of the BH3-only proteins. What seems to be clear is that all the BH3-only factors can interact, through their BH3 domains, with the anti-apoptotic members of the Bcl-2 family^21^. Some BH3-only proteins, such as Bim and Puma, bind all anti-apoptotic molecules with similar affinities, while others are more restricted. For example, Bad binds preferentially to Bcl-2, Bcl-xL, and Bcl-w, while Noxa shows a high affinity for Mcl-1 and Bfl-1^23^. Therefore, given that the ability to reach the apoptotic threshold would be determined not only by the mode of interaction but also by the levels of endogenous expression of pro- and anti-apoptotic factors, knowing the balance of expression of these factors and the BH3 members would be of importance to understand both physiological and pathological regulatory mechanisms.

Over the last few years, a new class of compounds called BH3-mimetics has been designed to inhibit anti-apoptotic Bcl-2 family members, leading to Bax and Bak activation. Also, the binding of BH3-mimetics to anti-apoptotic factors could trigger the release of activator BH3-only proteins from their target, which in turn could activate Bax and/or Bak directly^24–26^. Although BH3-mimetics have been shown to be effective in certain cancers, there are still considerable variations in the sensitivity of different cancer cells to these drugs^23^. To date, little is known about the relevance of Bcl-2 family members in GSC responsiveness to chemotherapeutic agents^27^. For this reason, in this study, we evaluated the role of Bcl-2 family members in the regulation of cancer stem cell survival in a panel of six GSC-ECLs using in vitro assays. Using BH3-mimetics (ABT-263, that targets Bcl-2, Bcl-xL and Bcl-w; WEHI-539, that solely targets Bcl-xL; and S63845 that inhibits Mcl-1)^24,28,29^, we determined that Bcl-xL and Mcl-1 are the most relevant Bcl-2 anti-apoptotic members for GSC-ECL survival and chemoresistance. Importantly, we found that the balance between the expression levels of Noxa and Mcl-1 varies between patient-derived GSC-ECLs and determines the sensitivity of these cells to Bcl-xL inhibitors. Therefore, our results position Noxa and Mcl-1 as key regulators of GSC sensitivity to Bcl-xL inhibitors.

## Results

### Expression of anti-apoptotic Bcl-2 family members in GSC-ECLs

**Figure 1:**
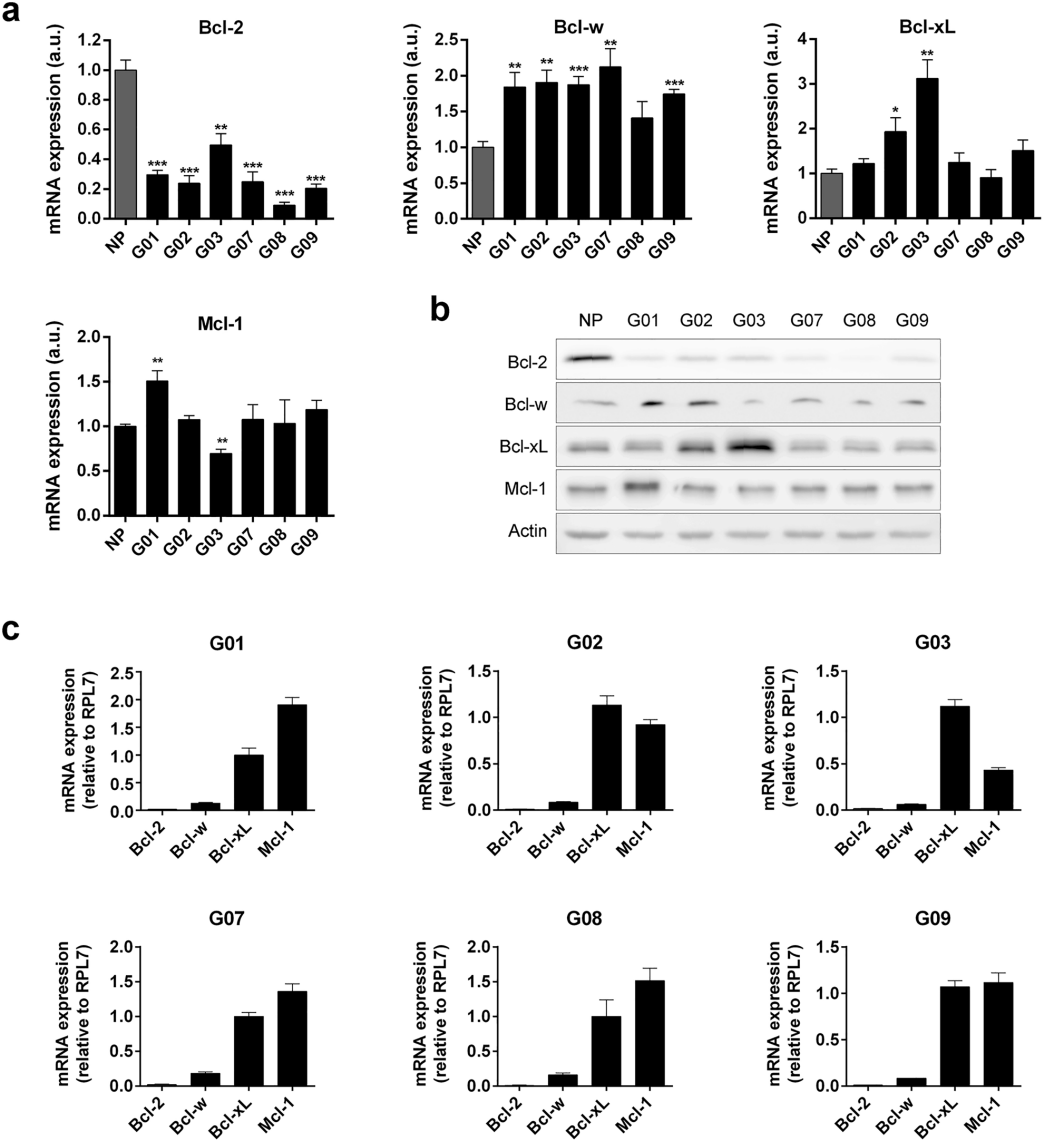
Expression levels of anti-apoptotic Bcl-2 family members in NP and GSC-ECLs. (**a**) mRNA expression levels by RT-qPCR. *rpl7* was used as normalizer. Graphs show mRNA expression fold change relative to NP. Bar charts represent the mean ± S.E.M. of three experiments. Student's *t-*test was used to detect significant differences between NP and each GSC-ECL. *P < 0.05, **P < 0.01, ***P < 0.001. a.u.: arbitrary units. (**b**) Protein levels were analyzed by Western blot. Actin was used as loading control. Representative blots of three independent experiments are presented. Full-length images are shown in Supplementary Figs. S11–S15. Bar graphs representing densitometric quantification of bands are depicted in Supplementary Fig. S1. (**c**) Transcriptional profile of anti-apoptotic Bcl-2 family members by RT-qPCR. *rpl7* was used as normalizer. Bars represent the mean ± S.E.M. of three experiments. For each cell line, the Y-axis scale was set based on the range of the values in the corresponding data set.

To evaluate the endogenous expression levels of anti-apoptotic factors, we performed RT-qPCR and Western blot analysis of Bcl-2, Bcl-w, Bcl-xL, and Mcl-1 in GSC-ECLs (named G01, G02, G03, G07, G08, and G09) and in untransformed neural progenitors (NP). We found that Bcl-2 expression is more abundant in NPs, while Bcl-w showed higher expression levels in GSC-ECLs. Although the abundance of Bcl-xL and Mcl-1 is similar between NP and GSC-ECLs, the G03 cell line exhibited higher expression levels of Bcl-xL, and G01 cell line showed increased Mcl-1 expression at both mRNA and protein levels (Fig. 1a,b and Supplementary Fig. S1). These differences in the expression levels of anti-apoptotic Bcl-2 family members between GSC-ECLs further support the well-known inter-tumor heterogeneity described in GBM. Finally, we also examined the transcriptional profile of these anti-apoptotic factors in each GSC-ECL. As shown in Fig. 1c, despite the variability in GBM, we found that Bcl-xL and Mcl-1 represent the most abundantly expressed anti-apoptotic factors in all tested GSC-ECLs while Bcl-w, followed by Bcl-2, exhibit the lowest expression levels.

### ABT-263 and WEHI-539 enhance GSC-ECL sensitivity to chemotherapeutic agents in a cell line-specific manner

As one of our main objectives was to evaluate the effectiveness of BH3-mimetics in sensitizing GSC-ECLs to chemotherapeutic agents, we defined concentrations of TMZ, CCNU and VCR at which they exert their primary effect but fail to induce excessive cell death. We established that the experimental conditions to achieve these requirements were 250 μM TMZ, 20 μM CCNU and 0.5 μM VCR for 48 h. At these conditions, we observed only a slight increase in cell death. Even so, TMZ and CCNU induced H2AX phosphorylation (γH2AX), indicative of DNA damage. VCR, on the other hand, led to the appearance of abnormally shaped cells, suggestive of the depolymerization of the microtubular network. Additionally, these chemotherapeutic drugs also caused the expected cell cycle arrest (Supplementary Fig. S2). Therefore, despite the lack of promoting extensive cell death in these conditions, these drugs were effective in exerting their mechanism of action.

**Figure 2:**
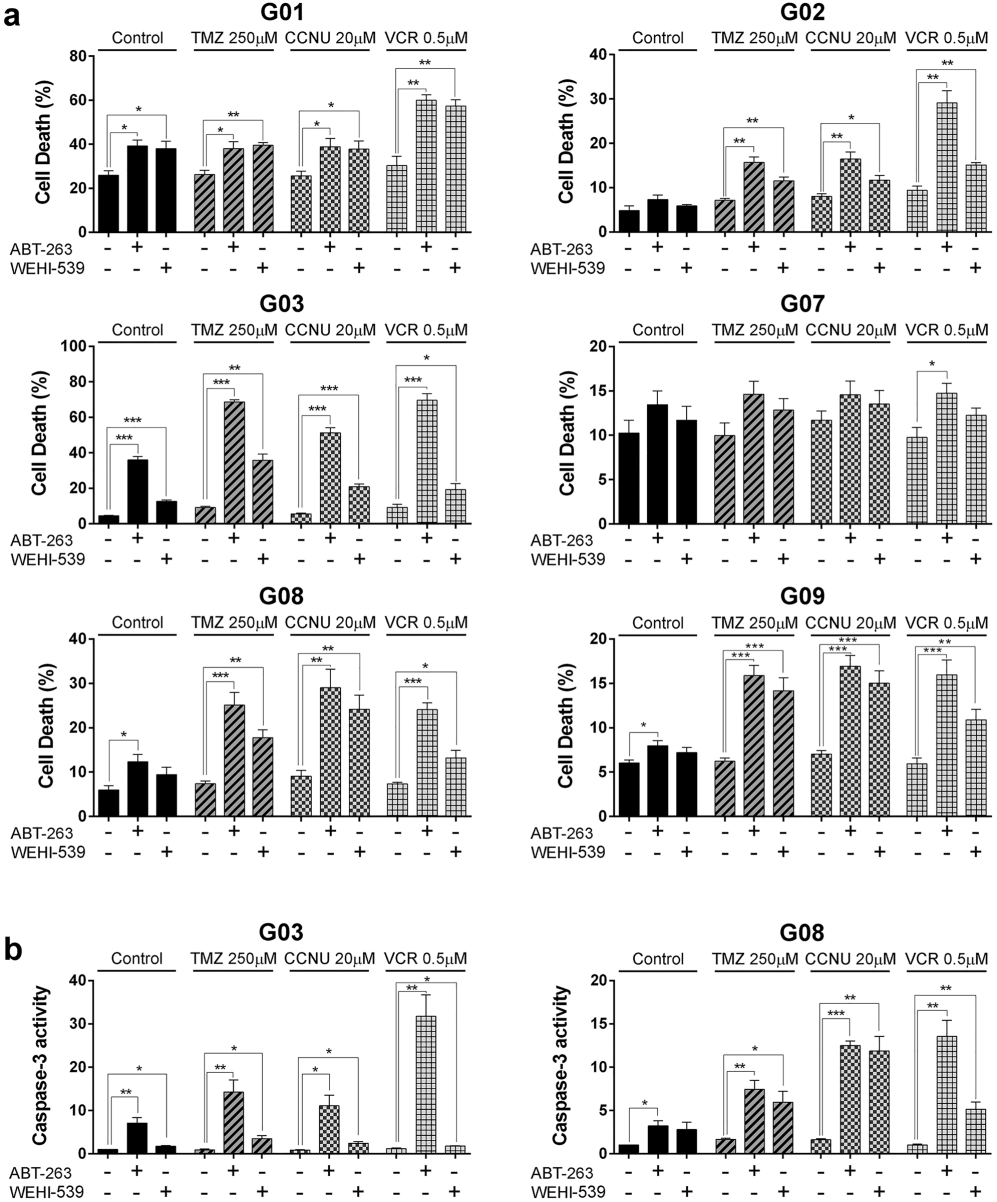
Effect of BH3-mimetics and chemotherapeutic agents on GSC-ECL viability. (**a**) Flow cytometric analysis of non-viable (PI^+^) cells 48 h after addition of chemotherapeutic agents in the presence or absence of ABT-263 (1 μM) or WEHI-539 (1 μM). Bar charts show the mean ± S.E.M. of three experiments. Student's *t-*test was conducted to detect significant differences. *P < 0.05, **P < 0.01, ***P < 0.001. (**b**) Caspase-3 activity in G03 and G08 cell line lysates was detected upon treatments by fluorometric assays. Graph shows caspase-3 activity fold change relative to untreated (control) cells. Bar charts show the mean ± S.E.M. of three independent experiments. Student's *t-*test was conducted to detect significant differences.*P < 0.05, **P < 0.01, ***P < 0.001. For each cell line, the Y-axis scale was set based on the range of the values in the corresponding data set.

As a first approach, we selected two BH3-mimetics, ABT-263 and WEHI-539, to target either a subset (Bcl-2, Bcl-w, and Bcl-xL) or individual (Bcl-xL) pro-survival proteins and evaluated their effect on GSC-ESCL cell viability. We characterized the sensitivity of two representative GSC-ECLs (G03 and G09) towards treatments with ABT-263 and WEHI-539 by dose-dependent experiments, both with and without TMZ (Supplementary Fig. S3) Based on this and on bibliography^28,30–33^, we selected a concentration of 1 μM for both inhibitors. In response to single treatments with these BH3-mimetics, G01, G03, G08, and G09 cell lines underwent a significant increase in cell death (Fig. 2a, black bars). To determine if these BH3-mimetics sensitize GSC-ECLs to chemotherapeutic agents, we also combined these BH3-mimetics with TMZ, CCNU, or VCR. In all cases, except for G07 cells, we found that the presence of ABT-263 or WEHI-539 significantly potentiated the extent of cell death compared to that triggered by each chemotherapeutic alone, although each cell line showed a differential sensitivity. In this sense, G03 cell line showed a considerably higher susceptibility to these BH3-mimetics than the other cell lines. On the contrary, G07 cell line showed resistance to these BH3-mimetics (Fig. 2a). Of note, except in the G01 cell line, ABT-263 treatment had a more pronounced effect on cell death than WEHI-539 in all tested conditions. However, this difference was strikingly higher in the G03 cell line (Fig. 2a).

Finally, to evaluate if the observed cell death is due to the activation of apoptosis, we assessed the activity of caspase-3 in two GSC-ECLs. As expected, we observed an increase in caspase-3 activity in treated cells, which could account for the observed changes in cell death (Fig. 2b).

### Bcl-xL is a major regulator of GSC-ECL viability

**Figure 3:**
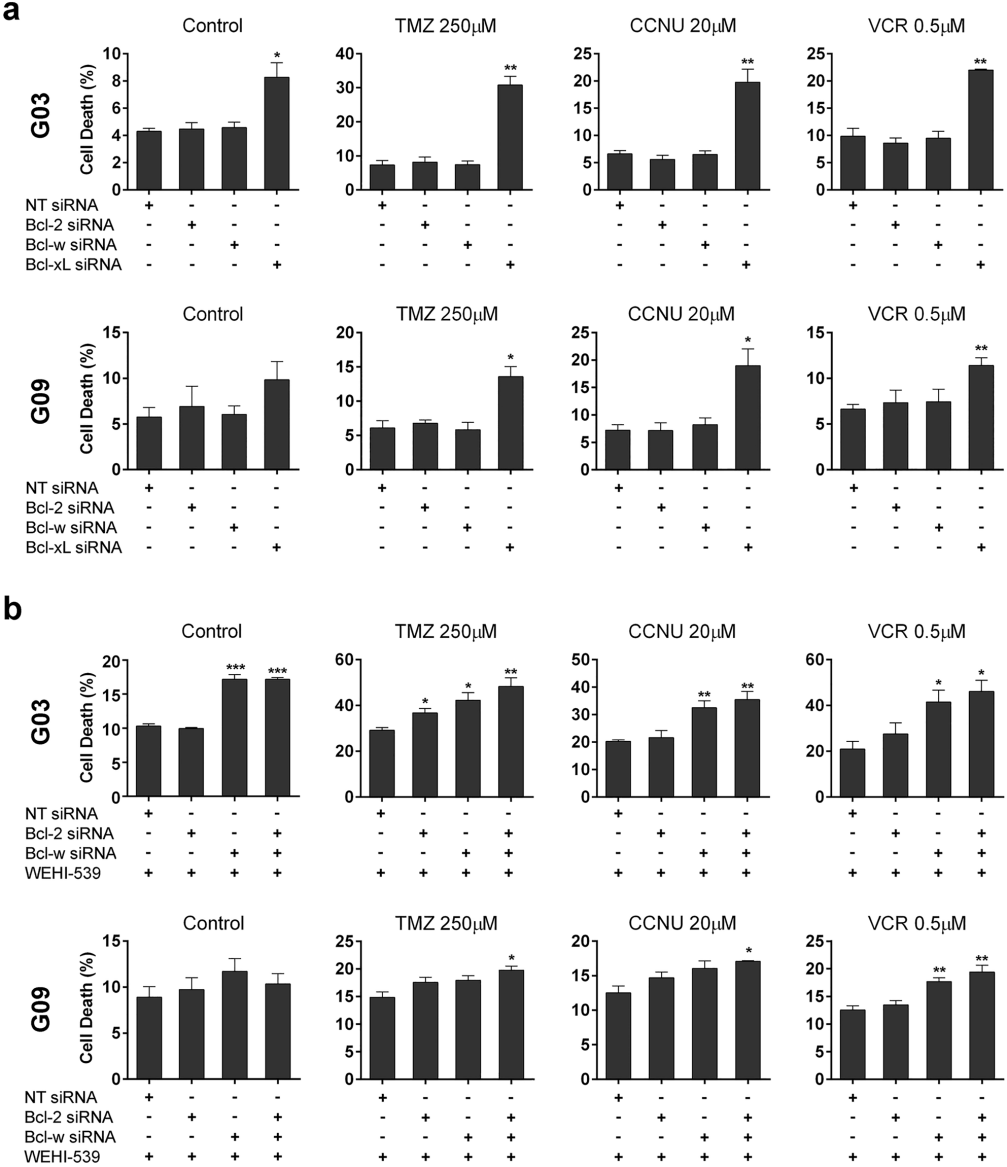
Involvement of Bcl-2, Bcl-w, and Bcl-xL in the control of GSC-ECL viability. (**a**) Percentage of cell death (PI^+^ cells) was determined by flow cytometry in G03 and G09 cells previously transfected with the indicated siRNA and treated or not with chemotherapeutic agents for 48 h. Non-targeting siRNA (NT siRNA) was used as a negative control. Each bar represents the mean ± S.E.M. of three independent experiments. Student's *t*-test was used to detect significant differences between NT-transfected cells and cells transfected with each specific siRNA. *P < 0.05, **P < 0.01, ***P < 0.001 (**b**) Percentage of cell death (PI^+^ cells) was determined by flow cytometry in G03 and G09 cells previously transfected with either NT, Bcl-2, Bcl-w, or Bcl-2 plus Bcl-w siRNAs, treated with WEHI-539 (1 μM) for 48 h and exposed or not to chemotherapeutic agents. Bar charts show the mean ± S.E.M. of three independent experiments. Student's *t*-test was used to detect significant differences between NT-transfected cells and cells transfected with each specific siRNA. *P < 0.05, **P < 0.01, ***P < 0.001. For each cell line, the Y-axis scale was set based on the range of the values in the corresponding data set.

To dissect the contributions of the anti-apoptotic factors targeted by ABT-263 (Bcl-2, Bcl-w, and Bcl-xL) on GSC-ECL viability, we conducted siRNA-mediated knockdowns for Bcl-2, Bcl-w, or Bcl-xL in GSC-ECLs (Supplementary Fig. S4). Only the silencing of Bcl-xL increased cell death (Fig. 3a and Supplementary Fig. S5). This result is consistent with the fact that Bcl-xL is one of the most abundant anti-apoptotic Bcl-2 family members and that its inhibition by WEHI-539 suffices to increase the sensitivity of the tested GSC-ECLs to antineoplastic drugs (Fig. 2a).

As previously shown, ABT-263 frequently led to higher cell death than WEHI-539, particularly in the G03 cell line (Fig. 2a). Thus, we hypothesized Bcl-2 and/or Bcl-w support cell viability when Bcl-xL is inhibited. To address this issue, we transfected cells with Bcl-2 and/or Bcl-w specific siRNAs in the presence of WEHI-539. Although we observed a trend towards increased cell death when these factors (principally Bcl-w) were silenced in the presence of WEHI-539 in all cell lines, the effect was much stronger in the G03 cells (Fig. 3b and Supplementary Fig. S6). Therefore, the higher efficacy of ABT-263 compared to WEHI-539 in G03 (Fig. 2a) would indicate a dependency of this cell line on Bcl-w and, less so, on Bcl-2, which may serve as a backup mechanism to survive when Bcl-xL activity is compromised. In accordance, in the G01 cell line, where ABT-263 and WEHI-539 caused a similar effect, we observed a low contribution of Bcl-2 and/or Bcl-w to cell survival (Supplementary Fig. S6).

### Noxa expression correlates with GSC-ECL sensitivity to ABT-263 and WEHI-539

**Figure 4:**
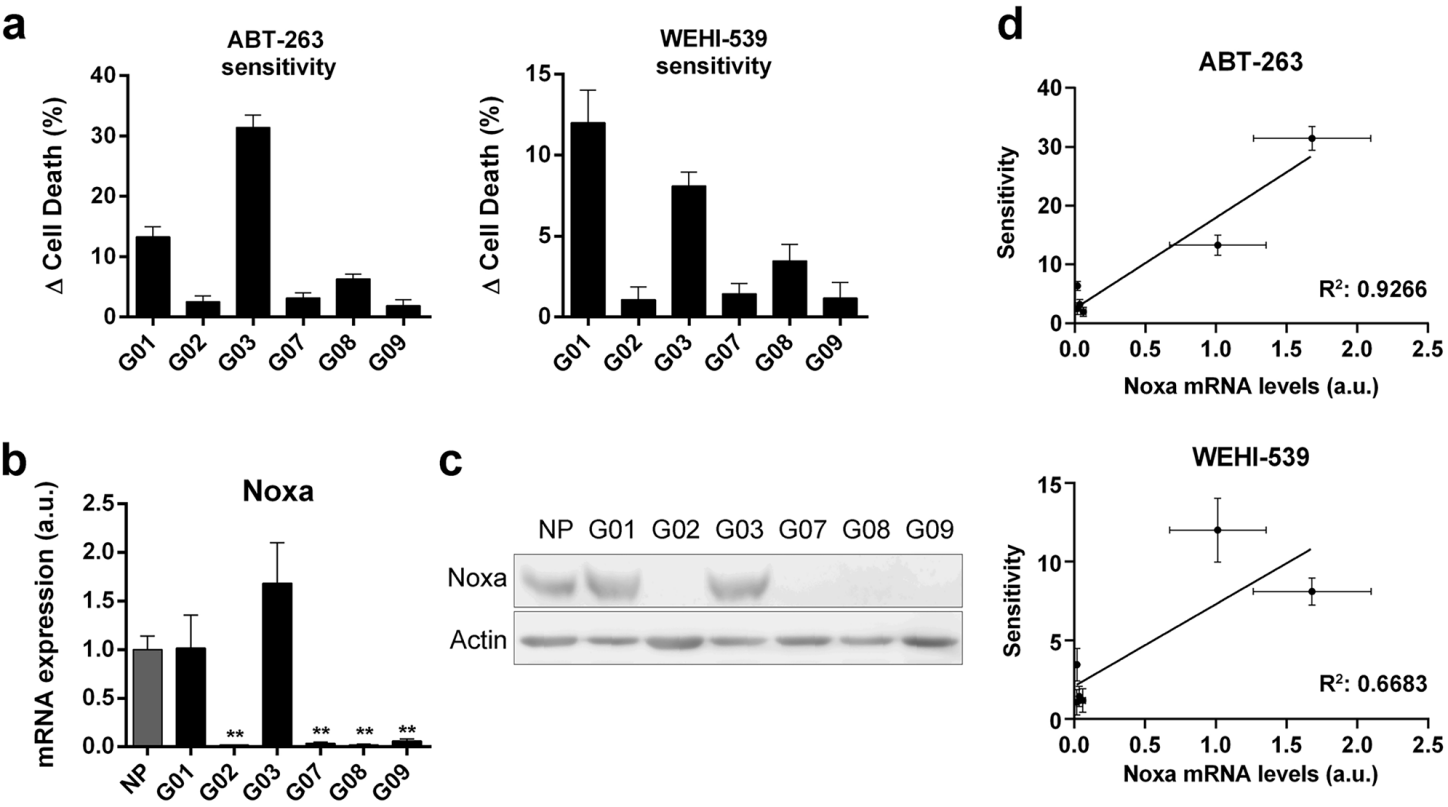
Noxa expression levels correlate with GSC-ECL sensitivity to BH3-mimetics targeting Bcl-xL. (**a**) Bar charts represent the differences (Δ) in the percentage of cell death (PI^+^ cells) calculated by subtracting the values of basal cell death from the ones obtained after exposure to ABT-263 or WEHI-539. (**b**) mRNA levels of Noxa were analyzed by RT-qPCR in NP and GSC-ECLs. *rpl7* expression was used as normalizer. Bars represent the mean ± S.E.M. of three experiments. Student's *t*-test was conducted to detect significant differences between NP and each GSC-ECL. *P < 0.05, **P < 0.01, ***P < 0.001. a.u.: arbitrary units. (**c**) Noxa protein levels were analyzed by Western blot. Actin was used as loading control. Full-length images are shown in Supplementary Fig. S16. Bar graphs representing densitometric quantifcation of bands are depicted in Supplementary Fig. S1. (**d**) Scatter plot showing relationship between Noxa mRNA levels and sensitivity to ABT-263 or WEHI-539. Simple linear regression visualized by black line. In both cases the slope is significantly different from zero (*p*-value < 0.01). R squared value represented on graph. For each cell line, the Y-axis scale was set based on the range of the values in the corresponding data set.

As previously described, each GSC-ECL displays a differential sensitivity to ABT-263 and WEHI-539 (Fig. 2a). To estimate the degree of BH3-mimetic sensitivity in each cell line, we calculated the difference (Δ) in the percentage of cell death by subtracting the cell death observed in untreated cells from the one observed in BH3-mimetic-treated cells (e.g. ABT-263-induced cell death—basal cell death). In both cases, G01 and G03 were the most sensitive cell lines to BH3-mimetic treatments (Fig. 4a).

Even though we have found that Bcl-xL inhibition has an important impact on cell death induction, we did not find any clear correlation between its abundance and GSC-ECL sensitivity to ABT-263 or WEHI-539. Thus, considering that BH3-mimetics emulate BH3-only proteins, we analyzed possible relationships between the expression of these factors and the sensitivity of GSC-ECLs to ABT-263 or WEHI-539. Notably, among the BH3-only factors (Supplementary Fig. S7) we found Noxa expression levels (Fig. 4b,c) significantly correlate with the degree of sensitivity to BH3-mimetics (Fig. 4d), being G01 and G03 the cell lines that exhibit the highest expression levels of this BH3-only (Fig. 4b,c). It is worth mentioning that although we find a correlation between Noxa expression and the sensitivity of GSC-ECLs towards Bcl-xL inhibitors, the analysis with a higher number of cell lines is needed to further confirm this finding.

### Impairment of Mcl-1 activity strongly potentiates WEHI-539-induced cell death in GSC-ECLs

**Figure 5:**
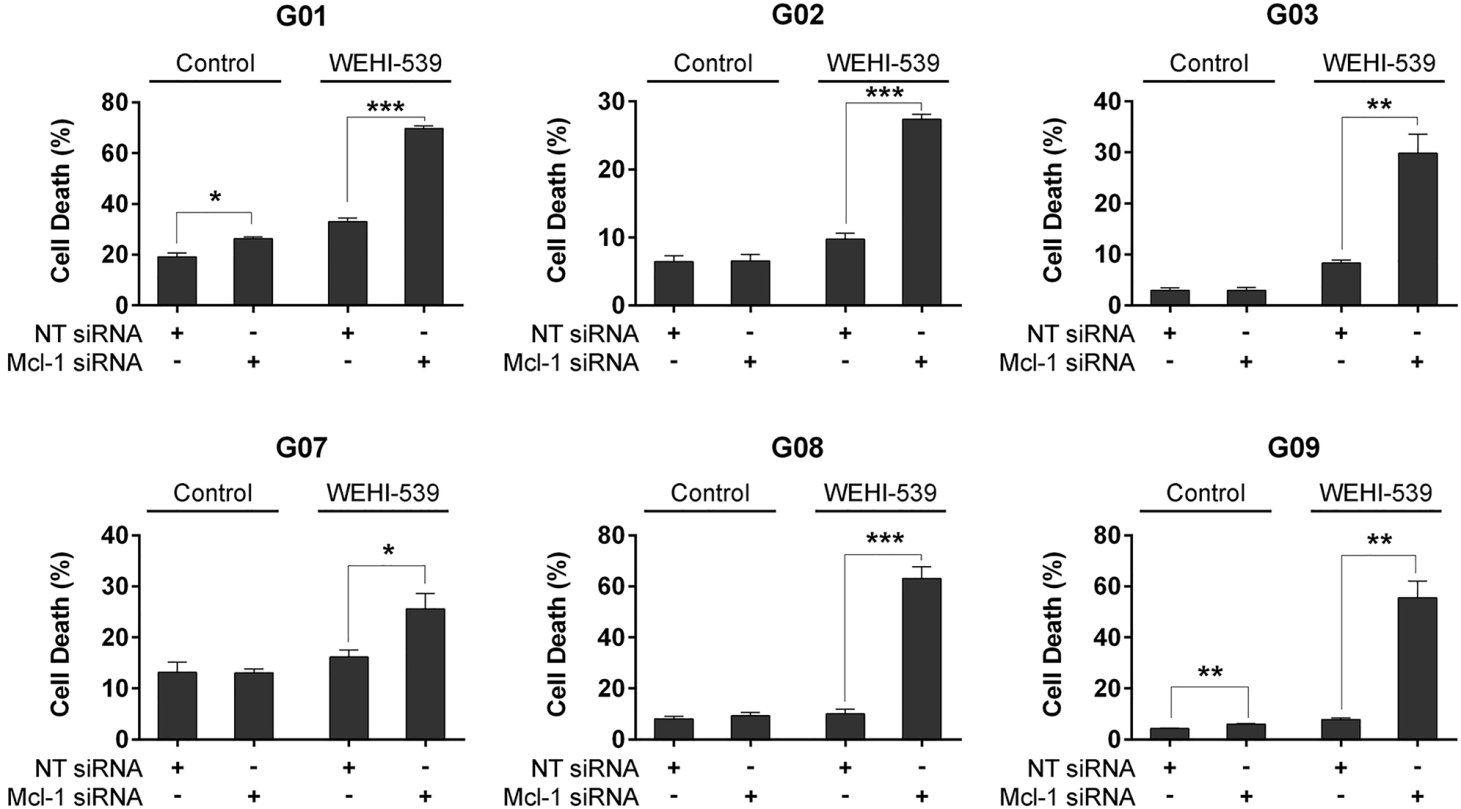
Silencing of Mcl-1 combined with Bcl-xL inhibition affects the viability of GSC-ECLs. Percentage of cell death (PI^+^ cells) was determined by flow cytometry in GSC-ECLs transfected with either NT or an Mcl-1 specific siRNA, and treated or not with WEHI-539 (1 μM) for 48 h. Each bar represents the mean ± S.E.M. of three experiments. Student's *t*-test was used to detect significant differences between NT-transfected cells and cells transfected with an Mcl-1 specific siRNA. *P < 0.05, **P < 0.01, ***P < 0.001. For each cell line, the Y-axis scale was set based on the range of the values in the corresponding data set.

At this point, we wondered whether the well-known Noxa binding partner, Mcl-1, was also responsible for the differential sensitivity of GSC-ECLs to ABT-263 and WEHI-539.To address this issue, we silenced this anti-apoptotic factor using a siRNA-specific sequence (Supplementary Fig. S4). In accordance with the high expression levels of Mcl-1, we found that this anti-apoptotic factor also contributes to GSC-ECL survival (Fig. 5). Even though Mcl-1 silencing alone slightly lowered the cell viability only in two GSC-ECLs, the combination with WEHI-539 was synergistic, inducing a notable increase of cell death in all tested cell lines (Fig. 5).

**Figure 6:**
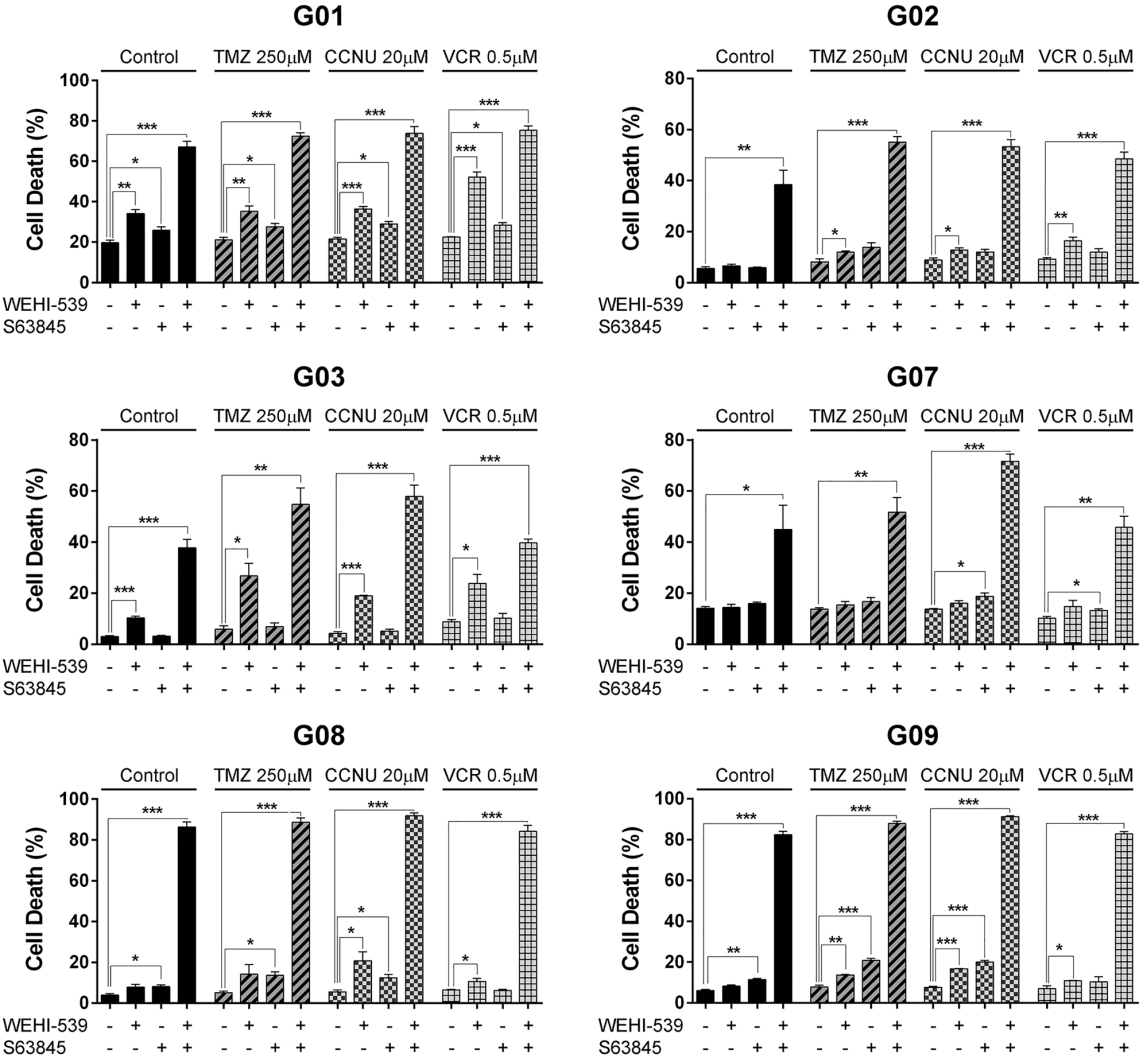
Combined inhibition of Mcl-1 and Bcl-xL strongly affects GSC-ECL viability. Percentage of cell death (PI^+^ cells) was determined by flow cytometry in GSC-ECLs treated for 48 h with chemotherapeutic agents, combined or not with S63845 (0.1 μM) and/or WEHI-539 (1 μM). Bar charts show the mean ± S.E.M. of three independent experiments. Student's *t*-test was conducted to detect significant differences *P < 0.05, **P < 0.01, ***P < 0.001. For each cell line, the Y-axis scale was set based on the range of the values in the corresponding data set.

To further evaluate the involvement of Mcl-1 in regulating GSC-ECL viability, first we estimated the sensitivity of G03 and G09 cells towards the Mcl-1 inhibitor S63845 by dose response experiments (Supplementary Fig. S3), and selected the 0.1 μM as the working concentration^34,35^. Then, we exposed cells to S63845 in the presence or absence of WEHI-539, combined or not with chemotherapeutic agents (Fig. 6). S63845 treatment as a single agent causes a slight increment in cell death. However, as occurred with Mcl-1 silencing, the combination of S63845 with WEHI-539 provokes a marked increase in cell death. Therefore the co-inhibition of the most abundantly expressed anti-apoptotic factors, Mcl-1 and Bcl-xL (Fig. 1c), causes substantial cell death in GSC-ECLs. Of note, in some cell lines (G08 and G09), the synergism between WEHI-539 and S63845 is so strong that triggered massive cell death even without chemotherapeutic agents.

Finally, to confirm that Noxa interacts with Mcl-1, we performed a co-immunoprecipitation assay. As expected, Noxa binds Mcl-1 in GSC-ECLs (Supplementary Fig. S8).

### Noxa expression determines sensitivity to ABT-263 and WEHI-539

To confirm that Noxa contributes to the induction of cell death triggered by these BH3-mimetics (either in the presence or absence of chemotherapeutic agents), we conducted gene silencing experiments using a Noxa specific-siRNA (Supplementary Fig. S4). For this purpose, we selected four cell lines that exhibited different basal expression levels of Noxa (G01 and G03 high; G02 and G09 low).

**Figure 7:**
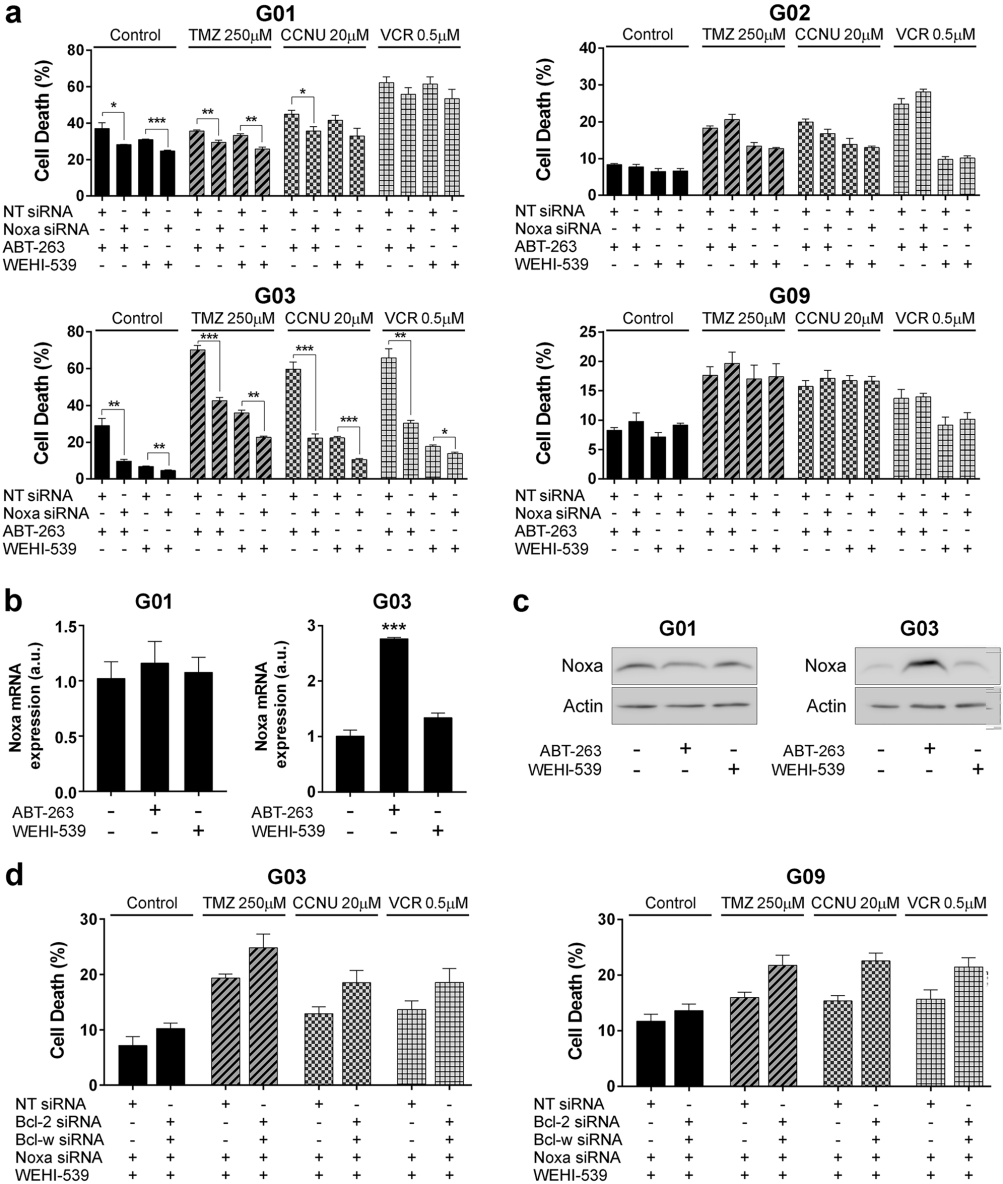
Noxa levels influence the sensitivity of GSC-ECL to BH3-mimetics. (**a**) Cells were transfected with either NT siRNA or Noxa specific siRNA and exposed 48 h to ABT-263 (1 μM) or WEHI-539 (1 μM), combined or not with chemotherapeutic agents. Percentage of cell death (PI^+^ cells) was determined by flow cytometry in GSC-ECLs. Bar charts show the mean ± S.E.M. of three independent experiments. Student's *t-*test was conducted to detect significant differences *P < 0.05, **P < 0.01, ***P < 0.001. (**b**) mRNA levels of Noxa were analyzed by RT-qPCR after 48 h of incubation with the indicated treatments. Bar charts show the mean ± S.E.M. of three independent experiments. Student's *t-*test was conducted to detect significant differences between untreated and treated cells. *P < 0.05, **P < 0.01, ***P < 0.001. (**c**) Protein levels of Noxa were analyzed by Western blot after 48 h of incubation with the indicated treatments. Actin was used as loading control. Full-length images are shown in Supplementary Figs. S22 and S23. Bar graphs representing densitometric quantifcation of bands are depicted in Supplementary Fig. S1. (**d**) G03 and G09 cells were transfected with either NT plus Noxa siRNAs or Bcl-2 plus Bcl-w plus Noxa siRNAs and exposed 48 h to WEHI-539 (1 μM). Percentage of cell death (PI^+^ cells) was determined by flow cytometry in GSC-ECLs. Bar charts show the mean ± S.E.M. of three independent experiments. For each cell line, the Y-axis scale was set based on the range of the values in the corresponding data set.

In the G03 cell line, down-regulation of Noxa significantly rescued cells from cell death induced by either ABT-263 or WEHI-539 (Fig. 7a). However, when cells were treated with chemotherapeutic agents in the absence of BH3-mimetics, silencing of Noxa did not affect cell death (Supplementary Fig. S9). Strikingly, in the G01 cell line, which also expresses relatively high basal levels of Noxa, its silencing promotes a much lesser decrease in cell death than in the G03 cell line (Fig. 7a). This could be explained by the fact that the G01 cell line expresses higher levels of Mcl-1 than the G03 cell line (Fig. 1). On the contrary, G02 and G09 cell lines showed no differences in cell death after silencing Noxa (Fig. 7). This result is in agreement with the fact that these cell lines express barely detectable levels of Noxa (Fig. 4b,c). As a whole, these findings demonstrate that Noxa expression levels not only correlate with sensitivity to Bcl-xL inhibition (Fig. 4d), but also contribute to the induction of cell death promoted by these BH3-mimetics.

To investigate whether BH3-mimetic treatments affect Noxa expression in the Noxa-dependent GSC-ECLs (G01 and G03), we measured mRNA and protein levels in untreated and treated cells. In the G03 cell line, ABT-263, but not WEHI-539, significantly induces Noxa expression (Fig. 7b,c). Notably, this induction coincides with the increase of cell death mediated by ABT-263 in this cell line (Fig. 2). Contrarily, in the G01 cell line, neither WEHI-539 nor ABT-263 induces Noxa expression (Fig. 7b,c).

Finally, as previously shown, the participation of Bcl-2 and Bcl-w in the G03 cell line is more relevant than in other cell lines (Fig. 3b and Supplementary Fig. S6), explaining the higher sensitivity of this cell line to ABT-263 than to WEHI-539 (Fig. 2a). Considering that Bcl-xL and Mcl-1 are the most relevant anti-apoptotic Bcl-2 family members in GSC-ECLs and that high levels of Noxa can inhibit Mcl-1, we hypothesized that in WEHI-539-treated G03 cells, Bcl-2 and Bcl-w become relevant due to the simultaneous inhibition of both Bcl-xL (due to WEHI-539) and Mcl-1 (due to the high levels of endogenous Noxa). To test this hypothesis, we evaluated the effect of silencing Bcl-2 and Bcl-w in WEHI-539-treated cells (as in Fig. 3b), but this time also silencing Noxa, therefore keeping Mcl-1 uninhibited. As shown in Fig. 7d, in WEHI-539-treated G03 cells, in a context of uninhibited Mcl-1, Bcl-2 and Bcl-w are no longer as relevant. As expected, in the G09 cell line, where the levels of Noxa are considerably lower, its silencing did not affect the contribution of Bcl-2 and Bcl-w to cell survival (Fig. 7d).

### The sensitizer role of Noxa depends on Mcl-1 activity in GSC-ECLs

**Figure 8:**
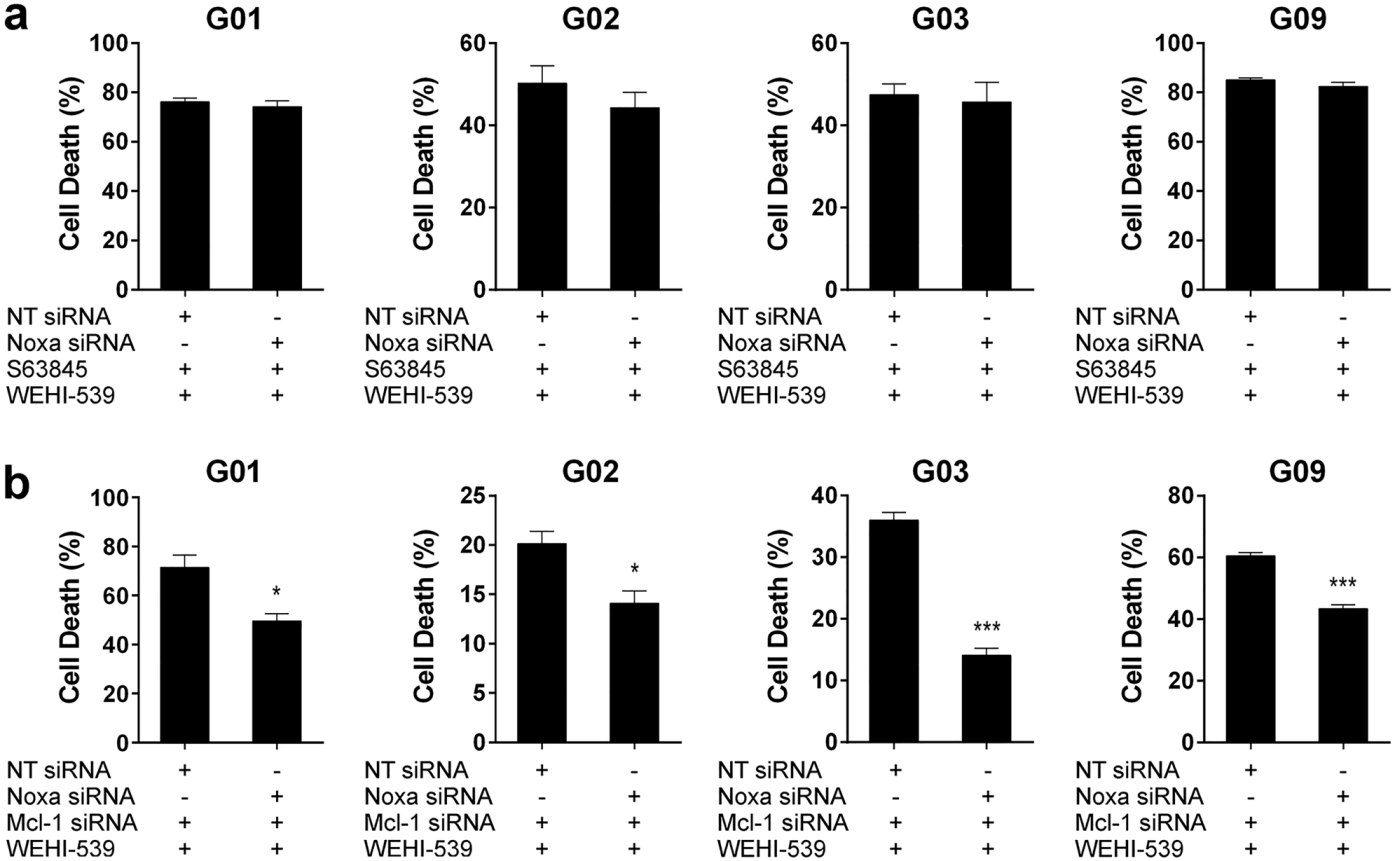
The sensitizer role of Noxa depends on Mcl-1 activity. (**a**) Percentages of cell death (PI^+^ cells) were determined by flow cytometry in cells transfected with either NT or Noxa siRNA and exposed 48 h to WEHI-539 (1 μM) plus S63845 (0.1 μM). Bar charts show the mean ± S.E.M. of three independent experiments. Student's *t-*test detected no significant differences. (**b**) Percentages of cell death (PI^+^ cells) were determined by flow cytometry in cells transfected with Mcl-1 siRNA combined with either NT or Noxa siRNAs, and exposed 48 h to WEHI-539 (1 μM). Bar charts show the mean ± S.E.M. of three independent experiments. Student's *t-*test was conducted to detect significant differences between untreated and treated cells. *P < 0.05, **P < 0.01, ***P < 0.001. For each cell line, the Y-axis scale was set based on the range of the values in the corresponding data set.

Having determined that Noxa influences GSC-ECL sensitivity to Bcl-xL inhibition, we explored the underlying mechanism of this response. To evaluate whether Noxa acts as an activator or a sensitizer BH3-only, we down-regulated its expression in a context in which Mcl-1 activity was inhibited. If Noxa behaves as an activator, the abrogation of Mcl-1 would increase cell death, partly by facilitating Noxa-mediated Bax/Bak oligomerization. Hence, silencing Noxa in this context would decrease cell death. On the contrary, if Noxa functions as a sensitizer, the lack of Mcl-1 activity would render Noxa irrelevant, as the only function of Noxa would be the neutralization of Mcl-1. As a consequence, Noxa silencing would not affect cell death. To discern between these two alternatives, we pharmacologically inhibited Mcl-1 (S63845) and Bcl-xL (WEHI-539) and evaluated the effect of silencing Noxa. Importantly, S63845 binds to Mcl-1 in the same binding groove that Noxa does^34^, promoting its displacement. As shown in Fig. 8a, the down-regulation of Noxa in the presence of S63845 did not decrease cell death. Therefore, these results indicate that Noxa acts as a sensitizer through its role as an Mcl-1 inhibitor in GSC-ECLs.

Having observed that the complete inhibition of Mcl-1 by S63845 renders Noxa irrelevant, we wondered if the partial decrease in the expression levels of Mcl-1 could also affect the relevance of Noxa. To find the answer, instead of inhibiting Mcl-1 with S63845, we silenced Mcl-1 and repeated the same experiments. Surprisingly, in this context, Noxa down-regulation caused a significant decrease in cell death in all tested cell lines (Fig. 8b), even in G02 and G09, in which Noxa silencing did not affect cell survival when Mcl-1 expression was unmodified (Fig. 7a). Importantly, in all tested cell lines, Noxa silencing led to a more pronounced decrease in cell death than that observed in cells with unmodified Mcl-1 expression (Fig. 8b and Supplementary Table S1). Therefore, Noxa becomes more relevant in the context of a diminished amount of Mcl-1. Of note, in all the studied cell lines, the basal expression levels of Mcl-1 transcripts far exceed those of Noxa (Supplementary Fig. S10). Thus, by lowering Mcl-1 expression, Noxa would now be able to bind and inactivate a higher proportion of this anti-apoptotic factor. Therefore, the relevance of Noxa as a sensitizer depends not only on its expression levels but also on those of Mcl-1, since when the expression of Mcl-1 decreases, the influence of Noxa on cell death increases.

## Discussion

Despite recent advances in cancer treatments, high-grade gliomas remain incurable. Therefore, there is an imperative need to find novel targets for the development of effective therapies. Recently, drugs targeting apoptotic regulators, such as BH3-mimetics, have emerged as novel therapies for the treatment of certain types of tumors^36^ based on findings that, in cancer, the apoptotic balance is often disturbed in favor of anti-apoptotic molecules. In high-grade gliomas the activity of Bcl-2 family members is usually deregulated; in particular, these tumors display increased expression of the anti-apoptotic factors Bcl-2, Bcl-xL, and Mcl-1, and decreased expression levels of the pro-apoptotic protein Bax^37^. In addition, higher overall levels of BH3-only protein expression correlate with survival rates in patients with GBM^38^. However, although the use of BH3-mimetics is being investigated in various cancer types, their application in gliomas remains under development^14,36^. The BH3-mimetics HA14-1, ABT-737, and ABT-263 promote apoptosis in GBM when used in combination with other pro-apoptotic agents^39–41^. Nevertheless, recent publications have presented conflicting data concerning the relevance of BH3-mimetics in glioma cell survival. One publication postulates that the inhibition of Mcl-1, but not the simultaneous inhibition of Bcl-2, Bcl-w, and, Bcl-xL with ABT-737 sensitizes glioma cells to TMZ^42^. Conversely, another publication proposes that simultaneous inhibition of Bcl-2, Bcl-w, and Bcl-xL by ABT-263, combined with the down-regulation of Mcl-1, significantly reduces the viability of glioma cell lines^41^. These studies were conducted using different glioma cell lines and experimental approaches, which could explain these discrepancies. In addition, to date, there are no reports that describe in depth the roles of individual Bcl-2 family members in patient-derived GSCs.

In this study, to cope with GBM inter-tumor heterogeneity, we used multiple patient-derived GSC-ECLs with diverse genetic and phenotypic backgrounds^9,10,43,44^ and analyzed the relative contribution of the anti-apoptotic Bcl-2 family members in GSC viability and chemoresistance. As expected, an overt variability between the GSC-ECLs was observed, mainly in terms of sensitivity to certain BH3-mimetics, such as ABT-263. However, despite this heterogeneity, in all of the analyzed GSC-ECLs, Mcl-1 and Bcl-xL were the most abundantly expressed anti-apoptotic factors and the most relevant, since their inhibition not only affected GSC-ECL viability but also sensitized them to different chemotherapeutics. After examining the expression levels of the BH3-only factors, we found a correlation between the expression of Noxa and the sensitivity of GSC-ECLs to Bcl-xL inhibitors. In certain GSC-ECLs, the high sensitivity observed to Bcl-xL inhibition could be explained by the high levels of Noxa that effectively inhibit the anti-apoptotic activity of Mcl-1. Therefore, in these GSC-ECLs, after treatment with Bcl-xL inhibitors (WEHI-539 or ABT-263), the two most relevant anti-apoptotic members of the Bcl-2 family (Bcl-xL and Mcl-1) would be simultaneously inhibited, leaving the cells more susceptible to different pro-apoptotic signals. Indeed, Noxa silencing only reduced sensitivity to ABT-263 or WEHI-539 in the GSC-ECLs that express the highest levels of Noxa (G01 and G03). However, in the G01 cell line, this effect was much less evident than in G03. This finding could be explained by the higher levels of Mcl-1 present in G01 cells, which may be high enough to attenuate the pro-apoptotic action of Noxa. In contrast, in other cell lines (G02 and G09), which exhibit lower levels of Noxa, the silencing of this BH3-only does not affect cell viability or chemoresistance. In these cases, the abundance of Mcl-1 greatly exceeds that of Noxa (compared to G01 and G03 cell lines), thus Noxa silencing would not significantly affect Mcl-1 activity. This higher amount of uninhibited Mcl-1 would also explain the higher resistance of these GSC-ECLs to Bcl-xL inhibition by ABT-263 or WEHI-539.

In addition, we found that Bcl-2 and Bcl-w exert secondary roles in cell survival, but only in particular molecular settings. For example, in the G03 cell line (which expresses high levels of Bcl-xL and high levels of Noxa), the activities of Bcl-2 and Bcl-w became relevant upon Bcl-xL inhibition, presumably because the endogenous levels of Noxa are sufficient to counteract the anti-apoptotic activity of Mcl-1, leaving the cells protected only by Bcl-2 and Bcl-w. Thus, in this cell line, this particular Bcl-2 family landscape would partly explain the notable differences between the extent of cell death triggered by ABT-263 (which inhibits Bcl-2, Bcl-w and Bcl-xL), and WEHI-593 (which only inhibits Bcl-xL). Moreover, in this cell line ABT-263, but not WEHI-539, significantly induces Noxa expression, further explaining why the G03 cell line is markedly sensitive to ABT-263 treatment. On the other hand, when compared to the G03 cell line, G01 cells express similar levels of Noxa but lower levels of Bcl-xL and higher levels of Mcl-1. The high levels of the latter would suffice to attenuate most of the cell death, leaving Bcl-2 and Bcl-w activities without relevance, explaining, at least in part, the similar sensitivity of G01 cells to ABT-263 and WEHI-539 treatments.

Finally, we observed that when Mcl-1 is inhibited, Noxa silencing has no impact on cell death, further supporting the concept that Noxa acts as a Mcl-1 inhibitor, that is to say, as a sensitizer BH3-only pro-apoptotic factor. Indeed, when Mcl-1 levels are lower, the pro-apoptotic function of Noxa as a sensitizer becomes prominent. Taken together, these results indicate that the sensitivity of GSCs to Bcl-xL inhibition would not depend on the mere expression of Noxa but on the balance between Noxa and Mcl-1 expression. In line with these findings, a recent study describes that photodynamic therapy combined with ABT-263 treatment increases Noxa/Mcl-1 ratio, promoting apoptosis in glioma cells^45^.

Despite the inter-tumor heterogeneity present in GBM, GSCs seem to share, as a common denominator, Bcl-xL and Mcl-1 as pivotal factors in mounting the anti-apoptotic response. These results, together with different studies that have shown that co-targeting Bcl-xL and Mcl-1 could be effective in melanoma, non-small cell lung cancer, epithelial tumors of the thymus, and prostate cancer^46–50^, would indicate that targeting these anti-apoptotic factors is a promising avenue in high-grade gliomas.

With this in vitro approach we have paved the way for further research into the roles of Noxa, Mcl-1, and Bcl-xL. To extend our observations, a more significant number of patient-derived cell lines and the use of different mouse models (including syngeneic tumor mouse models and those derived from genetically modified mice) are recommended to further assess the relevance of these findings. From a translational perspective, our results may help development of a model that predicts the effectiveness of including BH3-mimetics in chemotherapeutic treatments based on Noxa and Mcl-1 expression levels.

## Materials and methods

### Cell culture and treatments

The GSC-ECLs used in this study were previously isolated from human biopsies following relevant guidelines and national regulations. These cell lines have been characterized as described in previous reports^9,10,43,44^. Prior to their isolation, the use of these cell lines for research purposes have been approved by the Biomedical Research Ethics Committee “Comité de Ética en Investigaciones Biomédicas de la Fundación para la Lucha contra Enfermedades Neurológicas de la Infancia (FLENI)”. Written informed consent was received from each patient whose tumor tissue was used. NP cells were derived from human embryonic stem cells (WA09, provided by University of Wisconsin—Dr. J. Thomson. hPSCReg ID: WAe009-A)^51^. Both GSC-ECLs and NPs were grown in a serum-free medium consisting of Neurobasal supplemented with N2, B27, 20 ng/ml epidermal growth factor (EGF), 20 ng/ml basic fibroblast growth factor (bFGF) and plated onto Geltrex-coated plates (10 μg/ml) (all from Thermo Scientific, Rockford, IL, USA). BH3-mimetics and chemotherapeutic agents: ABT-263 (Santa Cruz Biotechnology, Santa Cruz, CA, USA), WEHI-539 (Genentech USA Inc, San Francisco, CA, USA), S63845 (Cayman Chemical, Ann Arbor, MI, USA), TMZ, VCR, and CCNU (Sigma, St. Louis, MO, USA).

### Reverse transcription polymerase chain reaction

For total RNA extraction, cells were dissociated with TRIzol reagent (Thermo Scientific, Rockford, IL, USA) following manufacturer's instructions. cDNA synthesis was carried out using MMLV reverse transcriptase (Promega, Madison, WI, USA). Quantitative RT-PCR assays were performed using SYBR® Green-ER™ qPCR SuperMix Universal (Thermo Scientific, Rockford, IL, USA). Primers used: Bcl-2 forward 5’-TATAACTGGAGAGTGCTGAAG-3’, reverse 5’-ACTTGATTCTGGTGTTTCCC-3’; Bcl-w forward 5’-TGGATGGTGGCCTACCTG-3’, reverse 5’-CGTCCCCGTATAGAGCTGTG-3’; Bcl-xL forward 5’-TGCGTGGAAAGCGTAGACAAG-3’, reverse 5’-GTGGGAGGGTAGAGTGGATGG-3’; Mcl-1 forward 5’-GGGCAGGATTGTGACTCTCATT-3’, reverse 5’-GATGCAGCTTTCTTGGTTTATGG-3’; Noxa forward 5’-ACCAAGCCGGATTTGCGATT-3’, reverse 5’-ACTTGCACTTGTTCCTCGTGG-3’; Bad forward 5’-ATGAGTGACGAGTTTGTGGAC-3’, reverse 5’-CGGGATGTGGAGCGAAGG-3’; Bid forward 5’-TGGGAGGGCTACGATGAG-3’, reverse 5’-GCTACGGTCCATGCTGTC-3’; Bik forward 5’-GCCGCCAGAGGAGAAATG-3’, reverse 5’-TCAGAGTCAGTCATGCCAAG-3’; Bim forward 5’-CCATGAGGCAGGCTGAACC-3’, reverse 5’-CATTCGTGGGTGGTCTTCGG-3’; Bmf forward 5’-AGGGTTGGGTTTCTCTAAG-3’, reverse 5’-ATGCTGACTGGCTATTCG-3’; Hrk forward 5’-GGCAGGCGGAACTTGTAG-3’, reverse 5’-TTACTCTCCACTTCCTTCTCG-3’; Puma forward 5’-GACCTCAACGCACAGTACGAG-3’, reverse 5’-AGGAGTCCCATGATGAGATTGT-3’; RPL7 forward 5’-AATGGCGAGGATGGCAAG-3’, reverse 5’-TGACGAAGGCGAAGAAGC-3’. PCR amplification was carried out using a StepOnePlus™ Real-Time PCR System (Applied Biosystems, Foster City, CA, USA).

### Immunofluorescence microscopy

The procedures for immunostaining were conducted as described previously^10^. Briefly, cells were rinsed with PBS and fixed in a 4% formaldehyde solution for 20 min. After three washes cells were permeabilized with 0.1% Triton X-100 in PBSA (PBS with 0.1% bovine serum albumin) with 10% normal bovine serum for 20 min, washed three times and incubated with the primary antibody in the same permeabilization buffer. Fluorescent-dye conjugated secondary antibodies (Thermo Scientific, Rockford, IL, USA) were used to detect the antigen/antibody complexes. Fixed cells were counterstained with DAPI and analyzed using a Nikon Eclipse TE2000-S inverted microscope. Images were acquired with a Nikon DXN1200F digital camera controlled by the EclipseNet software (version 1.20.0 build 61). The primary antibody used was α-γH2AX (cat # ab11175) (Abcam Inc., Cambridge, MA, USA).

### Western blotting

Cells were lysed in RIPA buffer supplemented with a protease inhibitor mix. Protein concentration was established using the Bicinchoninic Acid Protein Assay (Pierce™, Rockford, IL, USA). Equal quantities of protein were run on a 12% or a 15% polyacrylamide gel electrophoresis and transferred to PVDF membrane (Bio-Rad, Hercules, CA, USA). Membranes were blocked for 1 h with 5% skim milk in TTBS and incubated with primary antibodies for 16 h at 4 °C in blocking buffer (3% skim milk in TTBS). After incubation with the corresponding secondary antibody, protein bands were evidenced by chemiluminescence detection (SuperSignal West Femto System, Thermo Scientific, Rockford, IL, USA). Primary antibodies used: α-Actin (sc-1616), α-GAPDH (sc-47724); α-Bcl-2 (sc-7382); α-BclXS/L (sc- 634) (Santa Cruz Biotechnology, Santa Cruz, CA, USA); α-Bcl-w (cat # MA5-15076) (Thermo Scientific, Rockford, IL, USA); α-Mcl-1(cat # 94296); α-Noxa (cat # 14766) (Cell Signaling Technologies, Danvers, MA, USA). Secondary antibodies: a horseradish peroxidase-conjugated α-rabbit IgG; α-mouse IgG and α-goat IgG (Thermo Scientific, Rockford, IL, USA).

### Co-immunoprecipiation assay

Forty-eight hours after treatment cells were washed with ice-cold PBS containing Ca^2+^ and Mg^2+^, lysed with an NP-40 non-denaturing lysis buffer, and centrifuged at 14,000 × g at 4 °C for 15 min. Protein levels were quantified in the supernatant and 1 mg of protein was incubated with 10ul of Protein A/G Plus-Agarose (Santa Cruz Biotechnology, Santa Cruz, CA, USA) for 30 min at 4 °C on a rotator. After centrifugation at 3000 × g for 2 min at 4 °C the supernatant was incubated with 10ul anti-Noxa antibody (cat # 14,766) at 4 °C on a rotator. After three hours 10ul of Protein A/G Plus-Agarose was added, followed by two hours of incubation at 4 °C on a rotator. Samples were then centrifuged at 1000 × g for 1 min at 4 °C and the supernatant was discarded. The Agarose beads were washed three times with lysis buffer and then eluted to 40ul of SDS loading buffer by treatment for 10 min at 65 °C.

### Fluorometric caspase-3 activity assay

Caspase-3 activity in total GSC lysates was evaluated by EnzChek® Caspase-3 Assay Kit #2 (Molecular Probes Inc., Eugene, OR, USA) using rhodamine 110 bis-(N-CBZ-L-aspartyl-Lglutamyl-L-valyl-L-aspartic acid amide) (Z-DEVD–R110), according to the manufacturer's instructions. Briefly, the corresponding BH3-mimetics and/or chemotherapeutic agents were administered to the GSC-ECLs (5 × 10^5^cells/well in a 12-well plate). After 36 h, the cells were harvested and total cell lysates were prepared. Samples of the cell lysates were then seeded into 96-well plates were mixed with a reaction buffer and the plates were incubated for 30 min at room temperature protected from light. Then, the substrate of caspase-3 (25 µM Z-DEVD-R110), was added to each well and incubated for 1 h at 37 °C in the dark. Caspase-3 activity of cell extracts was determined by a fluorescence microplate reader (Fluoroskan Ascent FL, Thermo Fisher) using 496/520 nm excitation/emission wavelengths. The sample readings were calculated by subtracting the absorbance of the blank sample. Blank (no extract), negative control (extract from untreated cells).

### Cell transfection and RNA interference

2 × 10^5^ cells per well were plated on Geltrex-coated 6-well plates and transfected 24 h later with Lipofectamine RNAiMAX Transfection Reagent (Thermo Scientific, Rockford, IL, USA) and the corresponding siRNAs: Silencer Select Negative Control #2 (cat # 4390846); BCL2 siRNA (ID: 214532); BCL2L2 siRNA (ID: s1924); BCL2L1 siRNA (ID: 120717); MCL1 siRNA (ID: s8583); PMAIP1 siRNA (ID: s10709), all from Thermo Scientific, Rockford, IL, USA. siRNAs were added at final concentrations ranging between 5 and 50 nM.

### Flow cytometric analysis of cell viability and cell cycle distribution using propidium iodide (PI)

Cells were detached using Accutase (37 °C for 5 min). For viability assays, cells were then centrifuged at 200 × g for 3 min and resuspended in 300 μl of PBS. Cell suspensions were incubated with 3 μl of PI (1 mg/ml) for 5 min and immediately analyzed in a flow cytometer. Results were expressed as the percentage of PI^+^ cells (non-viable). For cell cycle distribution assays, cells were fixed in 70% ethanol, rehydrated in PBS, and treated with RNase A (1 mg/ml) and with PI (1 mg/ml). Fluorescence intensity was detected using a BD Accuri C6 flow cytometer (BD Biosciences, San Jose, USA).

## Supplementary Information

Supplementary Information.

## Data Availability

The datasets generated during and/or analyzed during the current study are available from the corresponding author on reasonable request.
