# Supplementary material for: Noxa and Mcl-1 expression influence the sensitivity to BH3-mimetics that target Bcl-xL in patient-derived glioma stem cells
Source: Sci Rep. 2022 Oct 22;12:17729. doi: 10.1038/s41598-022-20910-4 (PMC9587994; doi:10.1038/s41598-022-20910-4)
Supplement: Supplementary file 1 — Supplementary Information. [file 41598_2022_20910_MOESM1_ESM.pdf]

Densitometric analysis of western blot images

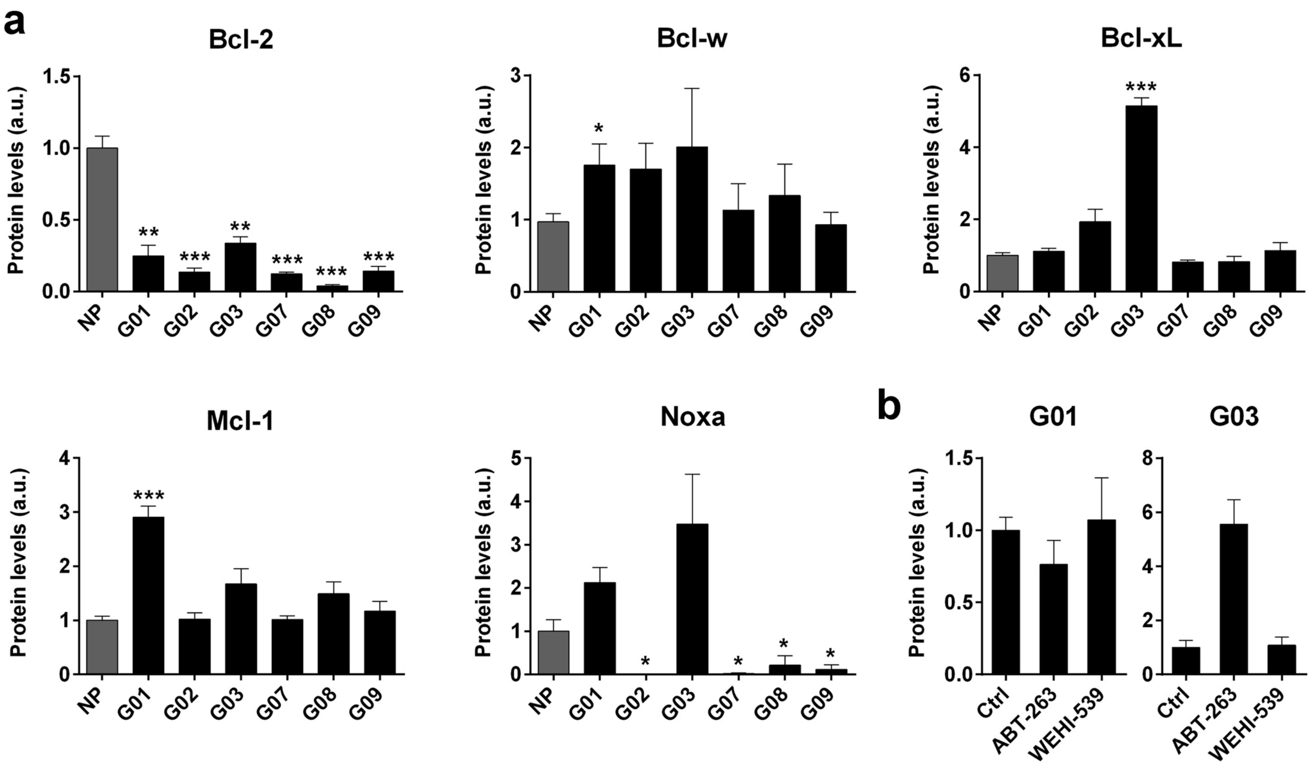

Supplementary Figure S1. Densitometric analysis of western blot images showed in Figures 1 and 4. Values were normalized to actin, and intensity is expressed as fold change relative to NP (a) or to untreated cells. Bar charts represent the mean ± S.E.M. of three experiments. Student's *t*-test was used to detect significant differences between NP and each GSC-ECL (a) and between untreated and treated cells (b). *P<0.05, **P <0.01, ***P<0.001. a.u.: arbitrary units.

G08 cell line response to chemotherapeutic agents

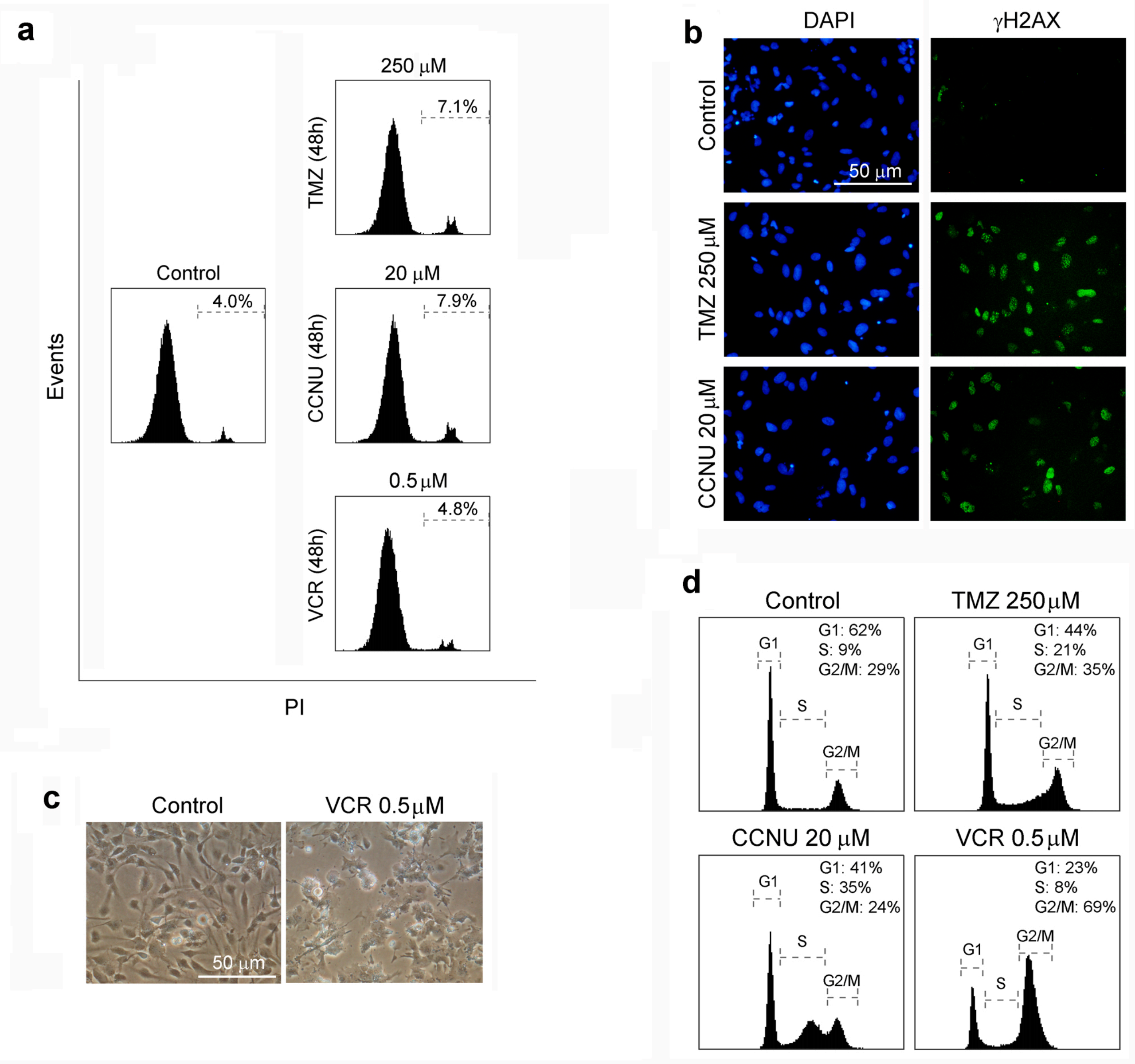

Supplementary Figure S2. G08 cell line response to chemotherapeutic agents. (a) Flow cytometric analysis of non-viable cells (PI^+) after 48 h treatment with TMZ, CCNU, or VCR. (b) Photomicrographs showing γH2AX immunofluorescence staining of TMZ- or CCNU-treated cells over a 48 h period. (c) Phase contrast images showing the morphology of G08 cells exposed to VCR for 48 h. (d) Flow cytometric analysis of cell cycle distribution in untreated or treated cells for 48 h

Differential sensitivity of G03 and G09 GSC-ECLs to BH3-mimetics

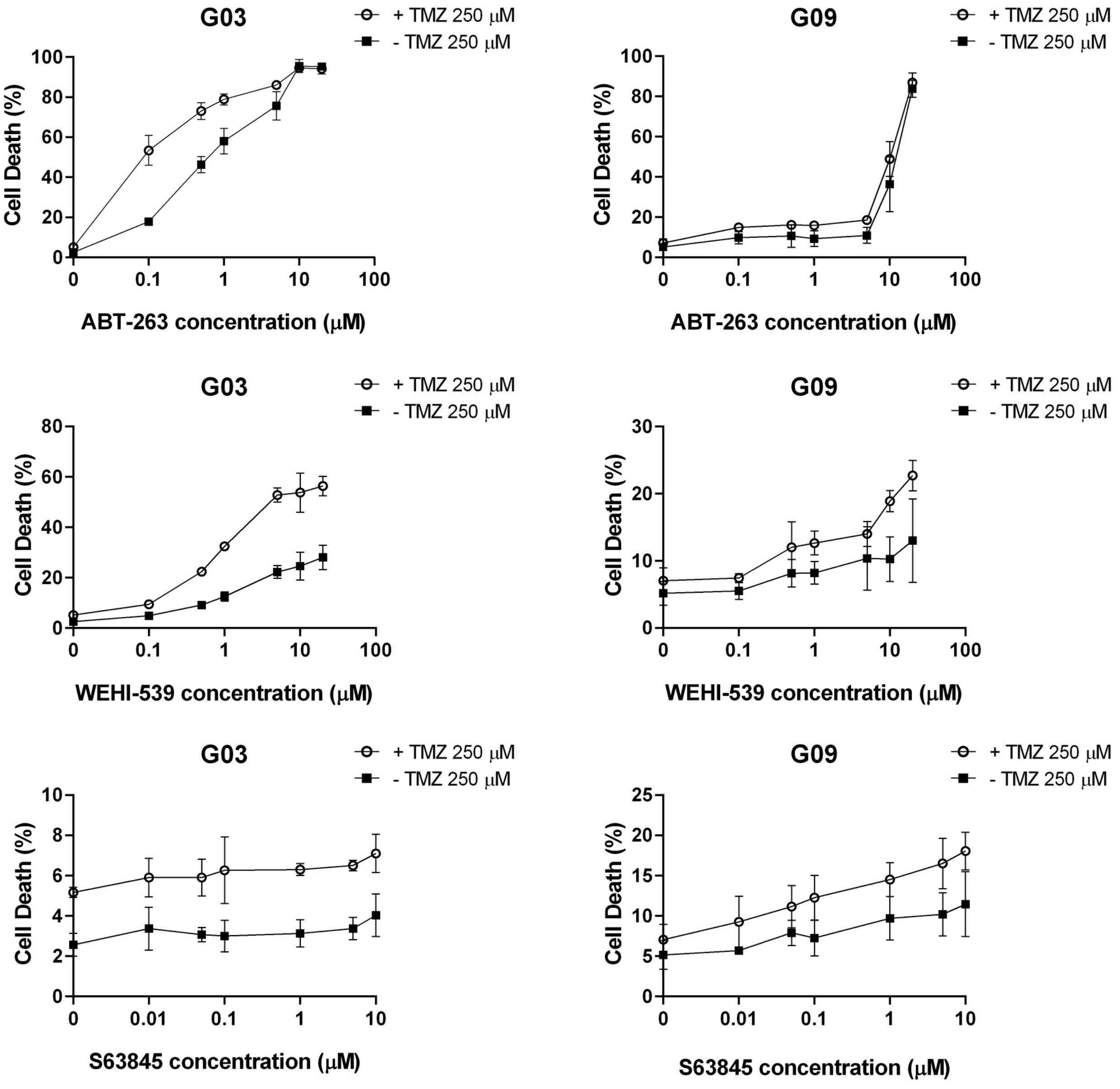

Supplementary Figure S3. Differential sensitivity of G03 and G09 GSC-ECLs to BH3-mimetics. Dose-response curves of three BH3-mimetics, combined or not with TMZ, were generated by flow cytometric analysis of non-viable (PI^+) cells 48 h after the corresponding treatments. The data are presented as the mean ± S.E.M. from three independent experiments.

Silencing efficiency of siRNAs in G03 cells

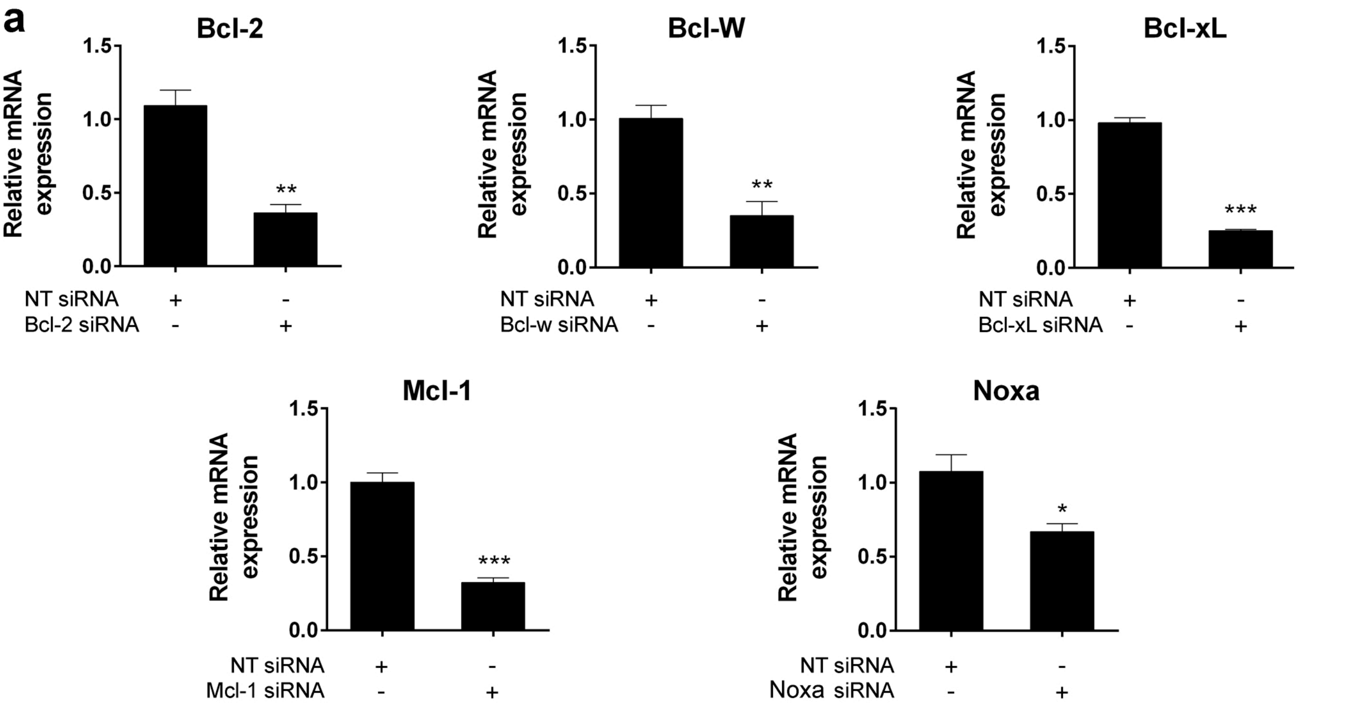

Supplementary Figure S4. Silencing efficiency of siRNAs in G03 cells. RT-qPCR analysis of mRNA levels 24 h after siRNA transfection. *rpl7* was used as normalizer. Student's *t* test was conducted to detect significant differences. *P<0.05, **P<0.01, ***P<0.001. a.u.: arbitrary units. Corresponding protein levels are shown in Supplementary Figures S17-S21.

Involvement of Bcl-2, Bcl-w, and Bcl-xL in the control of GSC-ECL viability

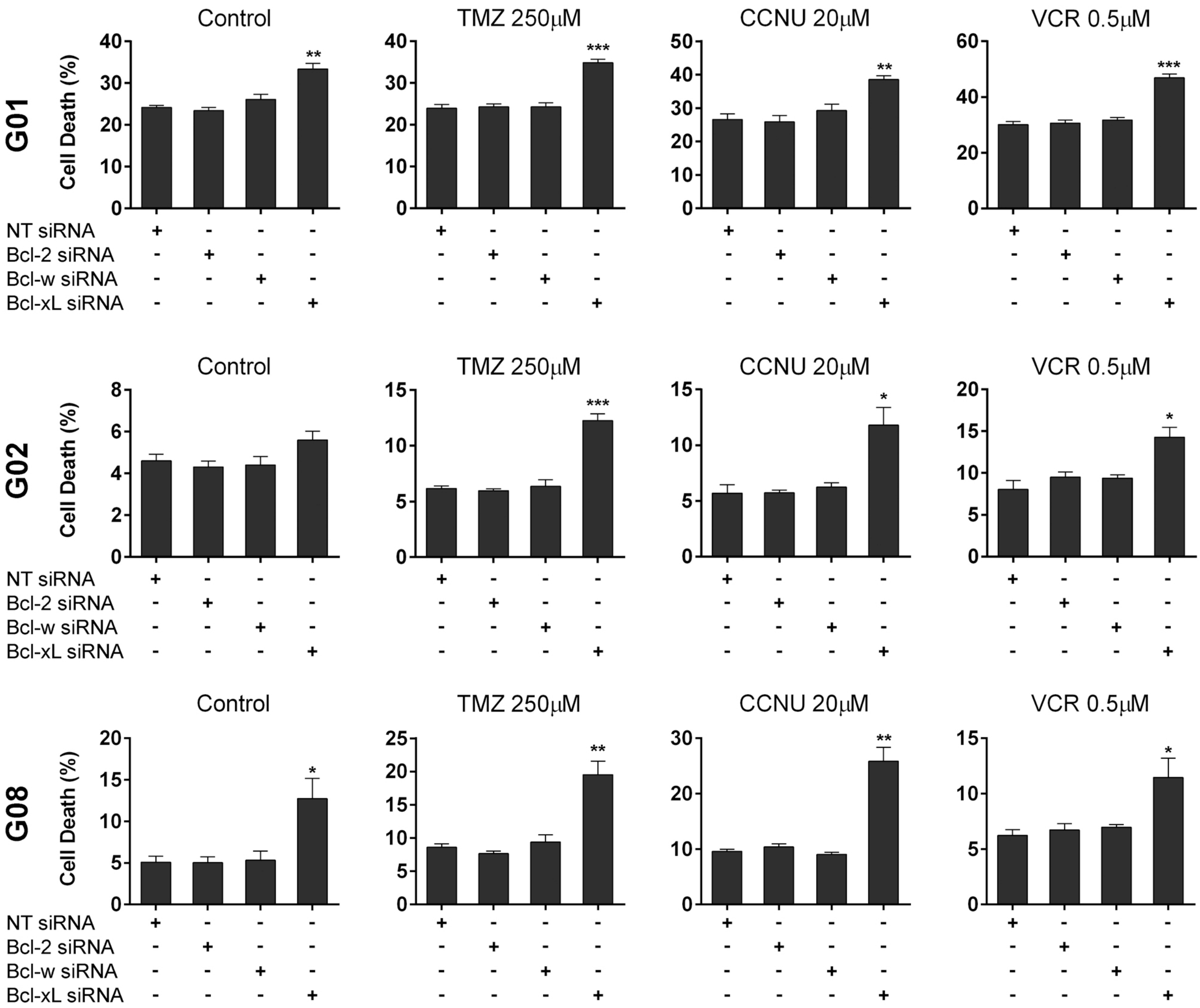

Supplementary Figure S5. Involvement of Bcl-2, Bcl-w, and Bcl-xL in the control of GSC-ECL viability. Percentage of cell death (PI^+ cells) was determined by flow cytometry in G01, G02, and G08 cells previously transfected with the indicated siRNA and treated or not with chemotherapeutic agents for 48 h. Non-targeting siRNA (NT siRNA) was used as a negative control. Each bar represents the mean ± S.E.M. of three independent experiments. Student's *t*-test was used to detect significant differences between NT-transfected cells and cells transfected with each specific siRNA. *P<0.05, **P<0.01, ***P<0.001.

Involvement of Bcl-2 and Bcl-w in GSC-ECL viability when Bcl-xL is inhibited

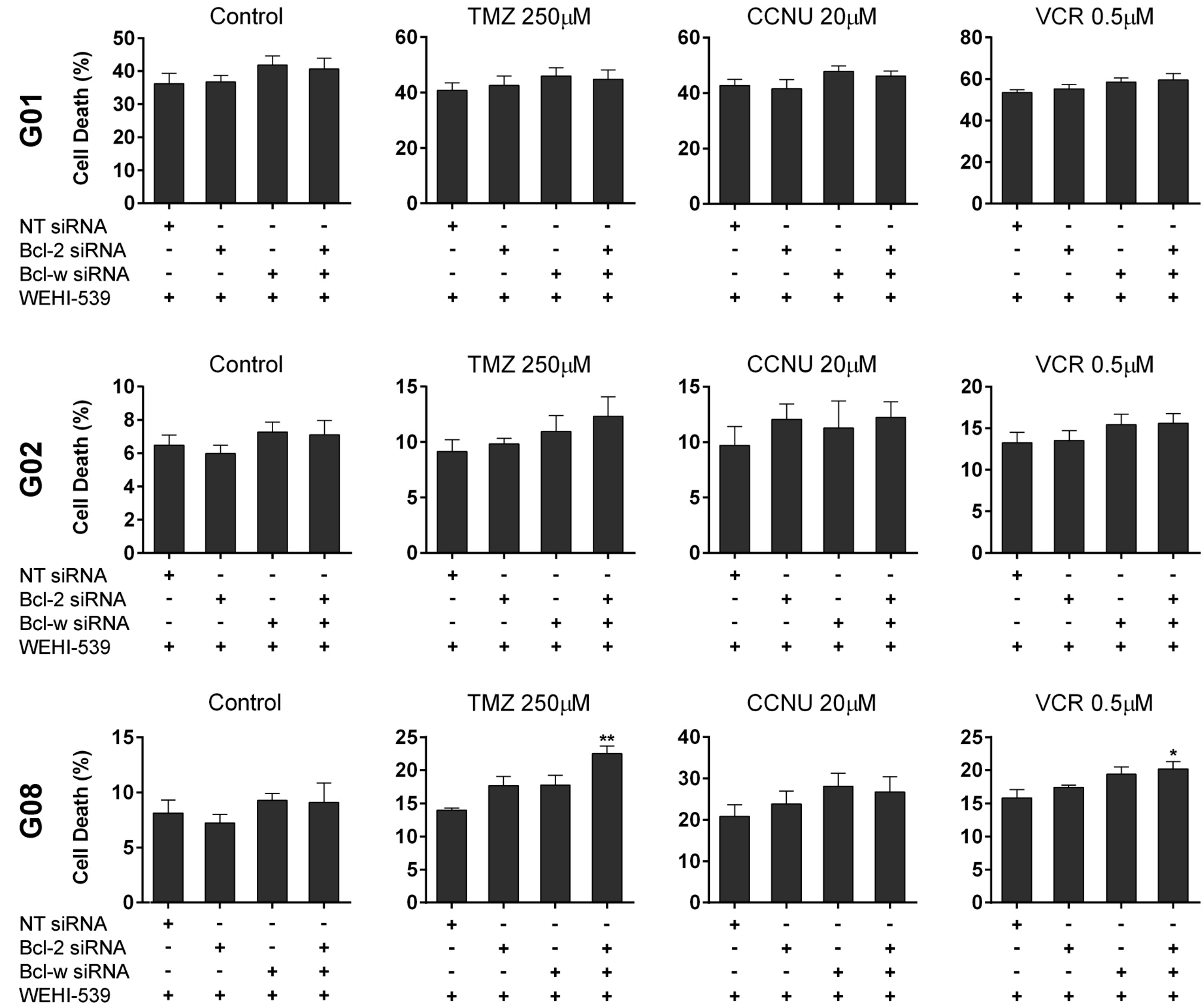

Supplementary Figure S6. Involvement of Bcl-2 and Bcl-w in GSC-ECL viability when Bcl-xL is inhibited. Percentage of cell death (PI^+ cells) was determined by flow cytometry in G01, G02, and G08 cells previously transfected with either NT, Bcl-2, Bcl-w or Bcl-2 plus Bcl-w siRNAs, treated with WEHI-539 (1 μM) for 48 h and exposed or not to chemotherapeutic agents. Bar charts show the mean ± S.E.M. of three independent experiments. Student's *t*-test was used to detect significant differences between NT-transfected cells and cells transfected with each specific siRNA. *P<0.05, **P<0.01, ***P<0.001.

Expression levels of BH3-only mRNA in GSC-ECLs

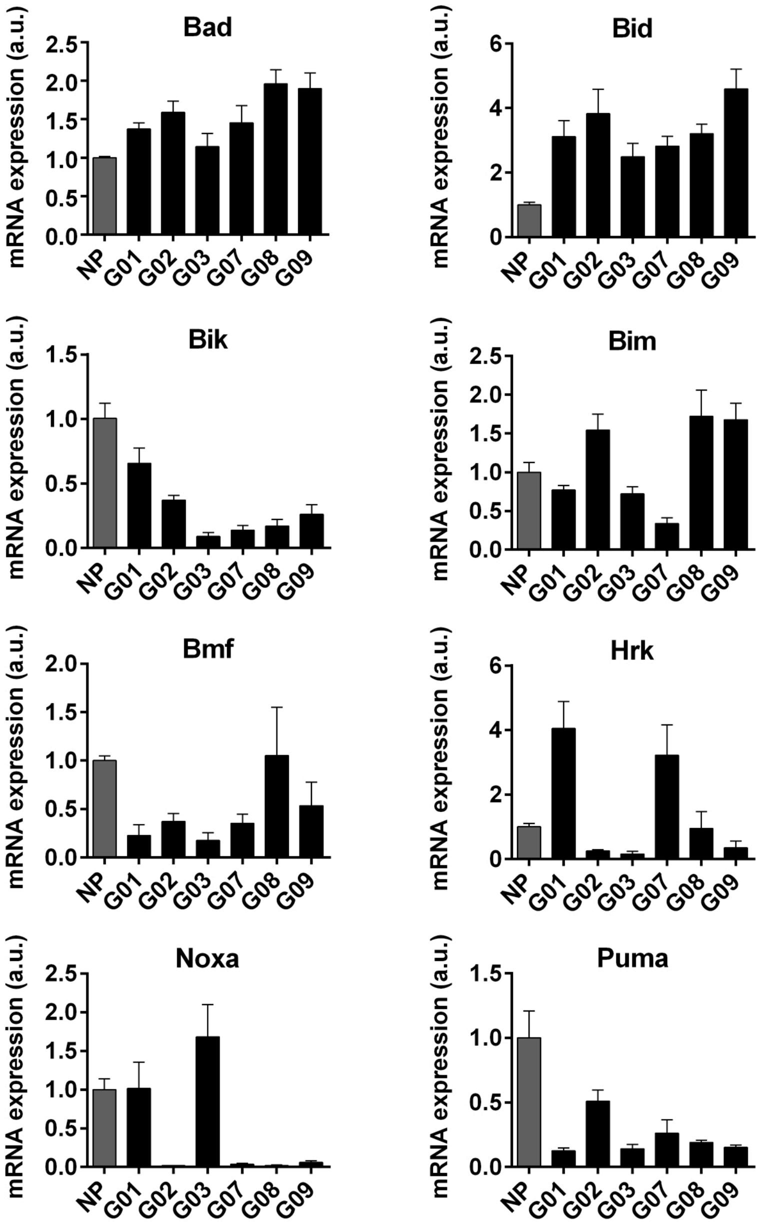

Supplementary Figure S7. Expression levels of BH3-only mRNA in GSC-ECLs. mRNA levels were analyzed by RT-qPCR. *rpl7* was used as normalizer.

Noxa co-immunoprecipitates with Mcl-1

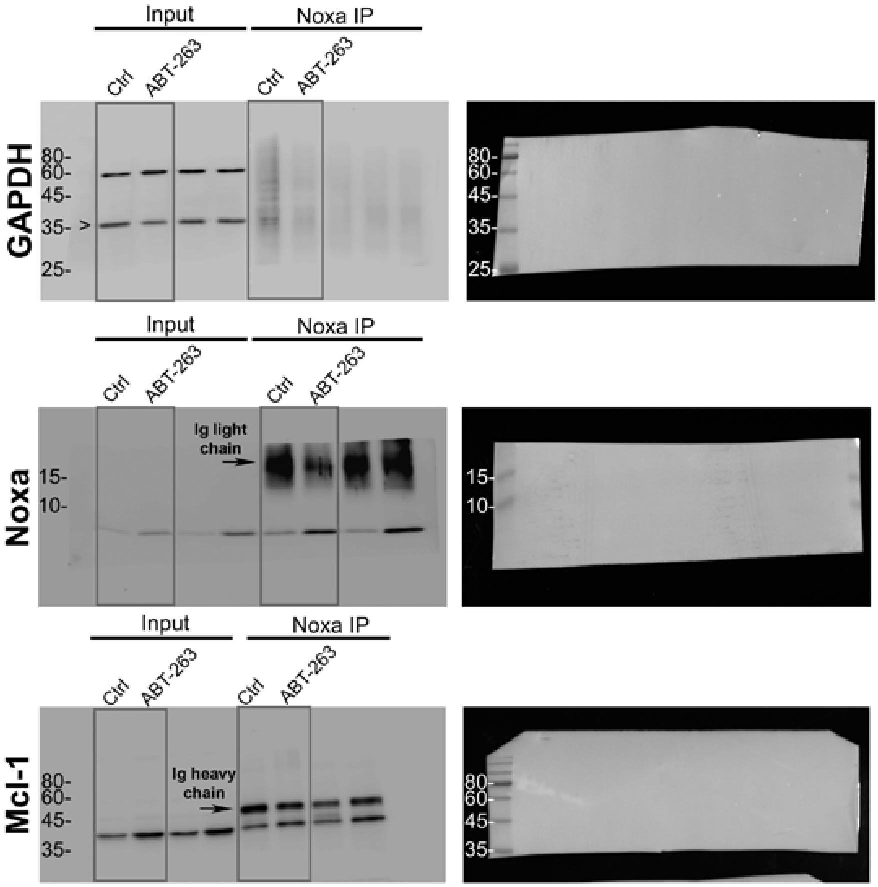

Supplementary Figure S8. Noxa co-immunoprecipitates with Mcl-1. Protein extracts from G03 cells were immunoprecipitated with anti-Noxa antibody. Western blots reveal the presence of Mcl-1 and Noxa in Noxa imunoprecipitated samples. GAPDH was used as the negative immunoprecipitation control. Relevant lanes are shown in boxes.

Noxa does not influence G03 cell line viability in response to chemotherapeutic agents

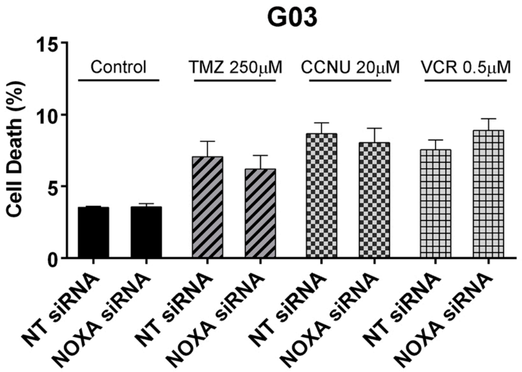

Supplementary Figure S9. Noxa does not influence G03 cell line viability in response to chemotherapeutic agents. Cells were transfected with the indicated siRNAs and exposed to the corresponding treatments. Flow cytometric analysis of non-viable (PI^+) cells was performed 48 h after addition of chemotherapeutic agents. Bar charts show the mean ± S.E.M. of three independent experiments.

Basal expression levels of Mcl-1 transcripts considerably exceed those of Noxa

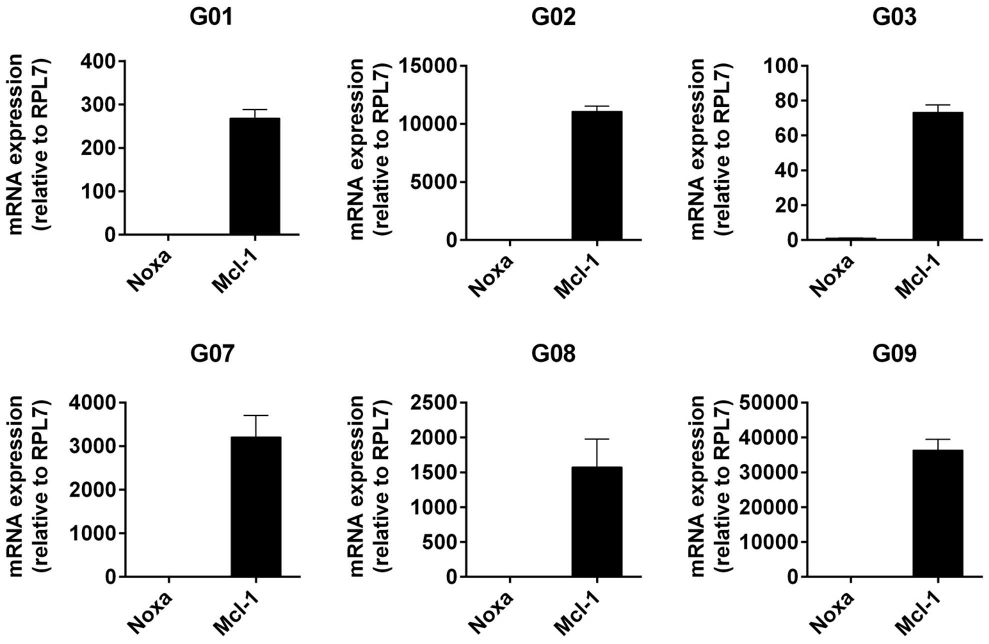

Supplementary Figure S 10. Basal expression levels of Mcl-1 transcripts considerably exceed those of Noxa. Expression levels of Noxa and Mcl-1 by RT-qPCR in each GSC-ECLs. *rpl7* was used as normalizer. Bars represent the mean ± S.E.M. of three experiments.

Full lenght Western blot images of Actin protein levels

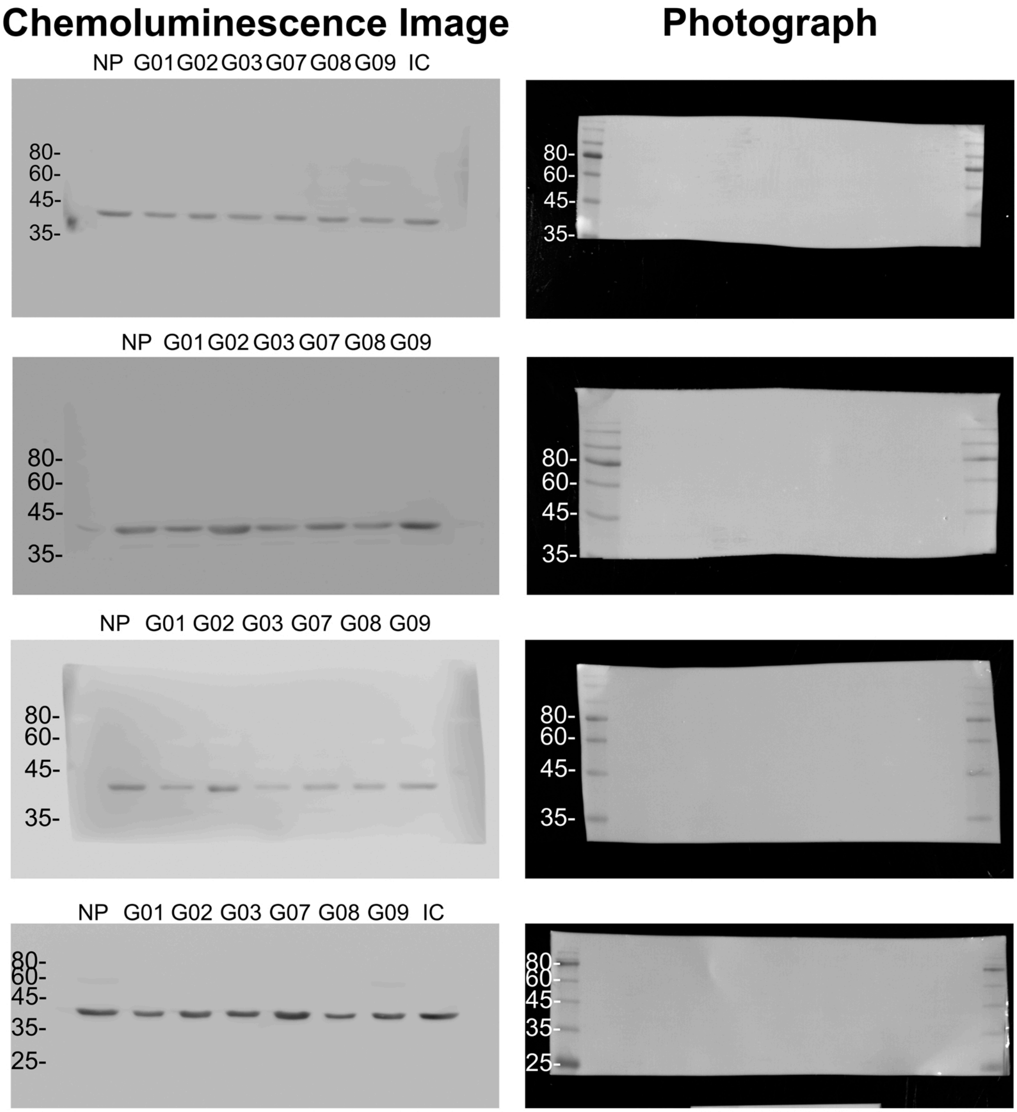

Supplementary Figure S11. Full length Western blot images of Actin of Figures 1 and 4. Blue Plus IV Protein Marker (Trans) was used as a protein size marker. IC: Internal Control.

Full lenght Western blot images of Bcl-2 protein levels

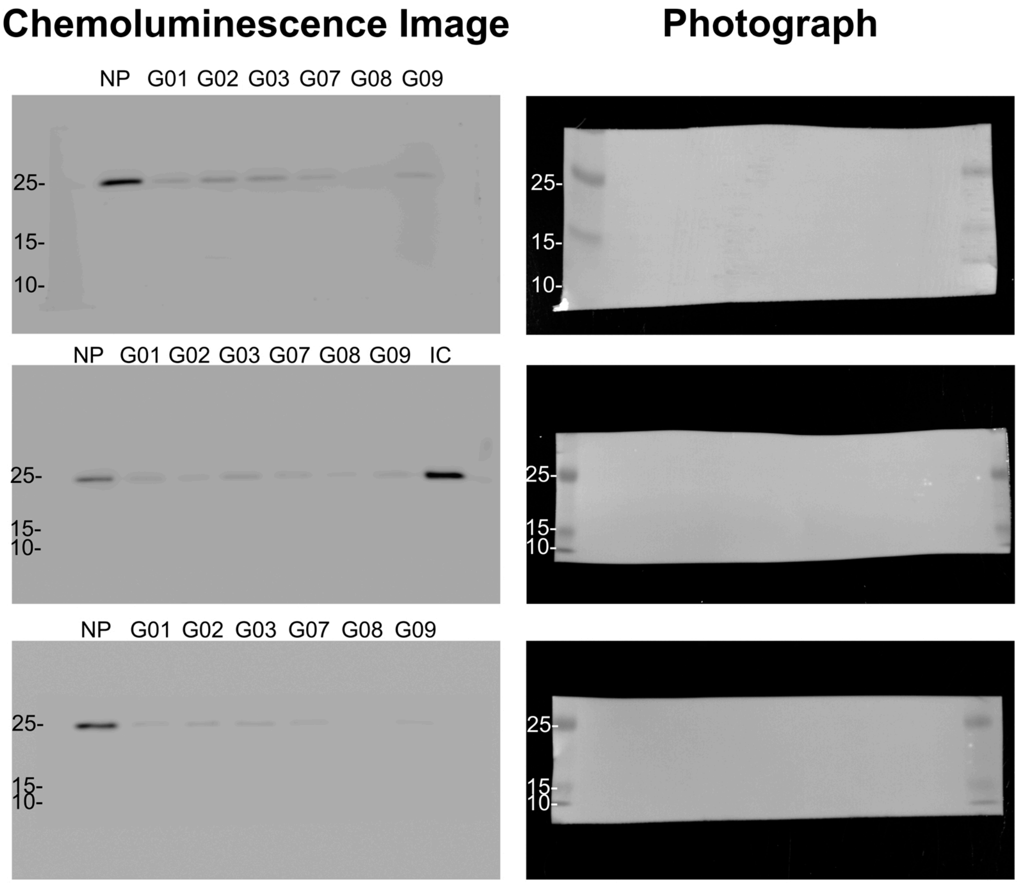

Supplementary Figure S12. Full length Western blot images of Bcl-2 of Figure 1. Blue Plus IV Protein Marker (Trans) was used as a protein size marker. IC: Internal Control.

Full lenght Western blot images of Bcl-w protein levels

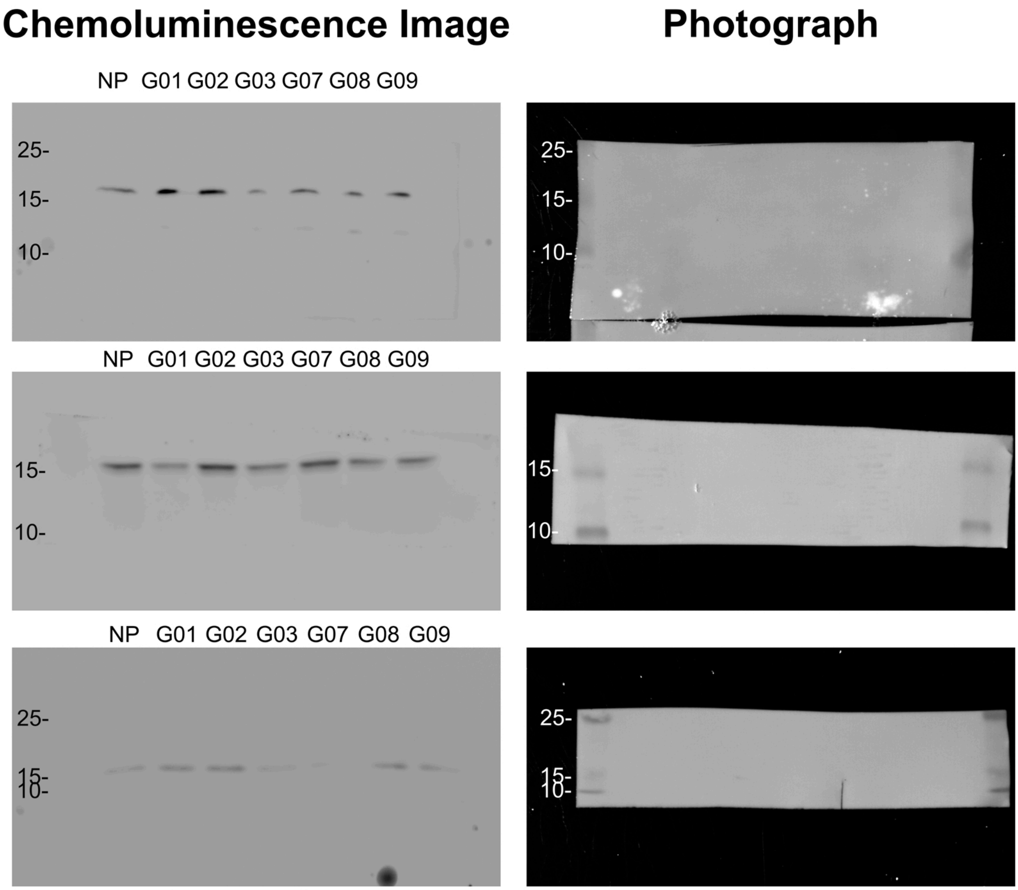

Supplementary Figure S13. Full length Western blot images of Bcl-w of Figure 1. Blue Plus IV Protein Marker (Trans) was used as a protein size marker.

Full lenght Western blot images of Bcl-xL protein levels

Chemoluminescence Image **Photograph**

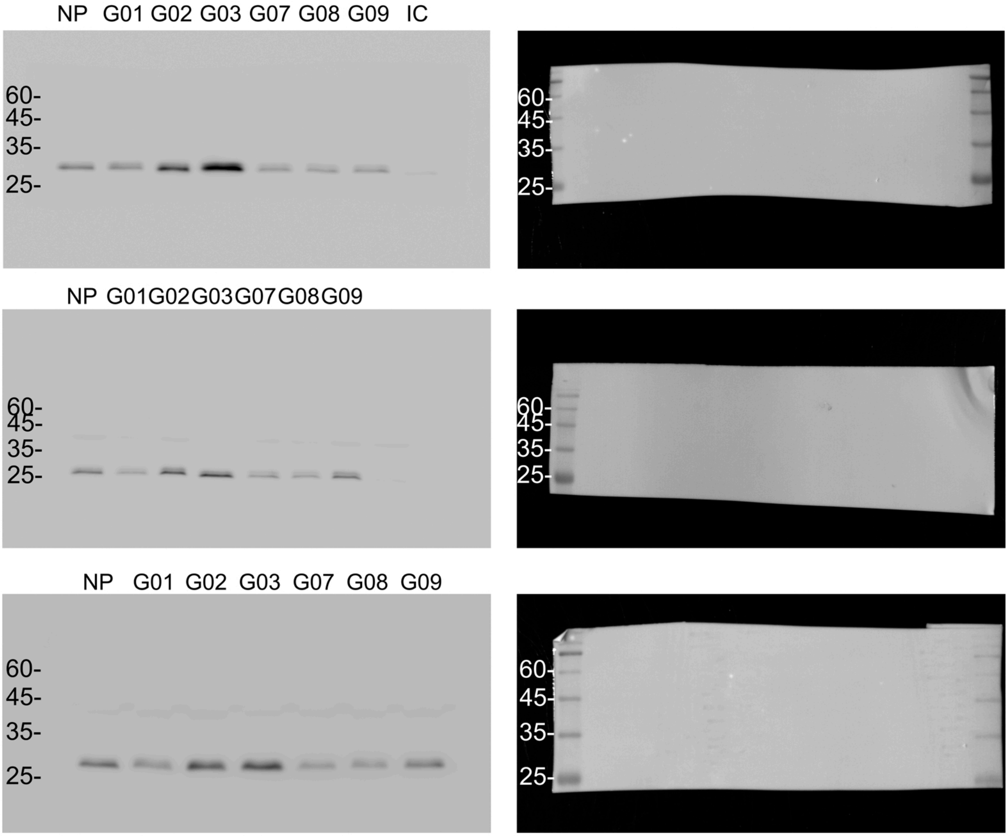

Supplementary Figure S14. Full length Western blot images of Bcl-xL of Figure 1. Blue Plus IV Protein Marker (Trans) was used as a protein size marker. IC: Internal Control.

Full lenght Western blot images of Mcl-1 protein levels

Chemoluminescence Image Photograph

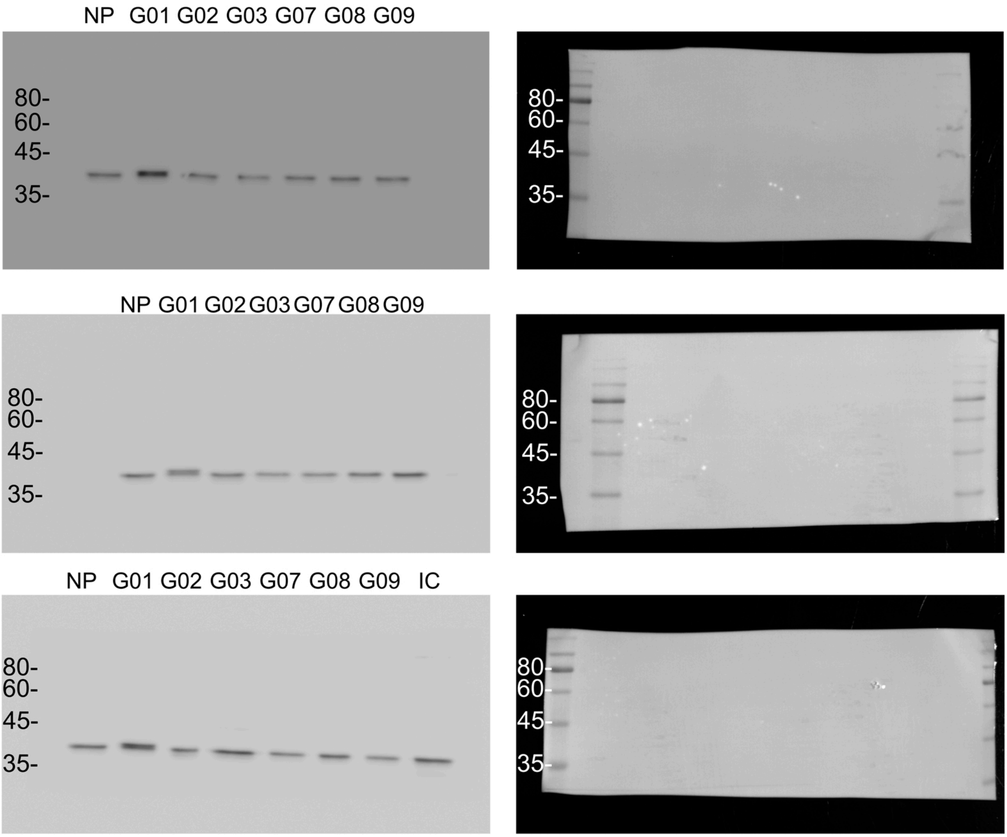

Supplementary Figure S15. Full length Western blot images of Mcl-1 of Figure 1. Blue Plus IV Protein Marker (Trans) was used as a protein size marker. IC: Internal Control.

Full lenght Western blot images of Noxa protein levels

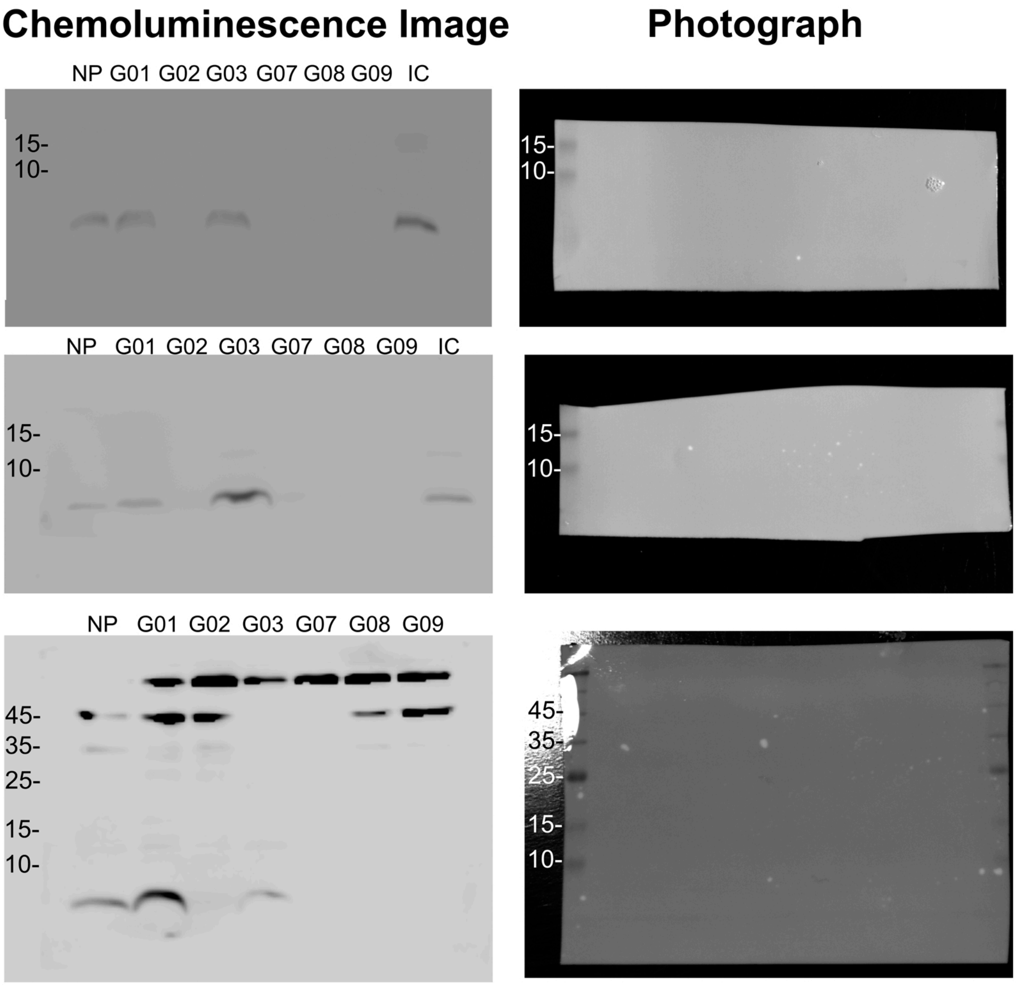

Supplementary Figure S16. Full length Western blot images of Noxa of Figure 4. Blue Plus IV Protein Marker (Trans) was used as a protein size marker. IC: Internal Control.

Full lenght Western blot images of Bcl-2 silencing

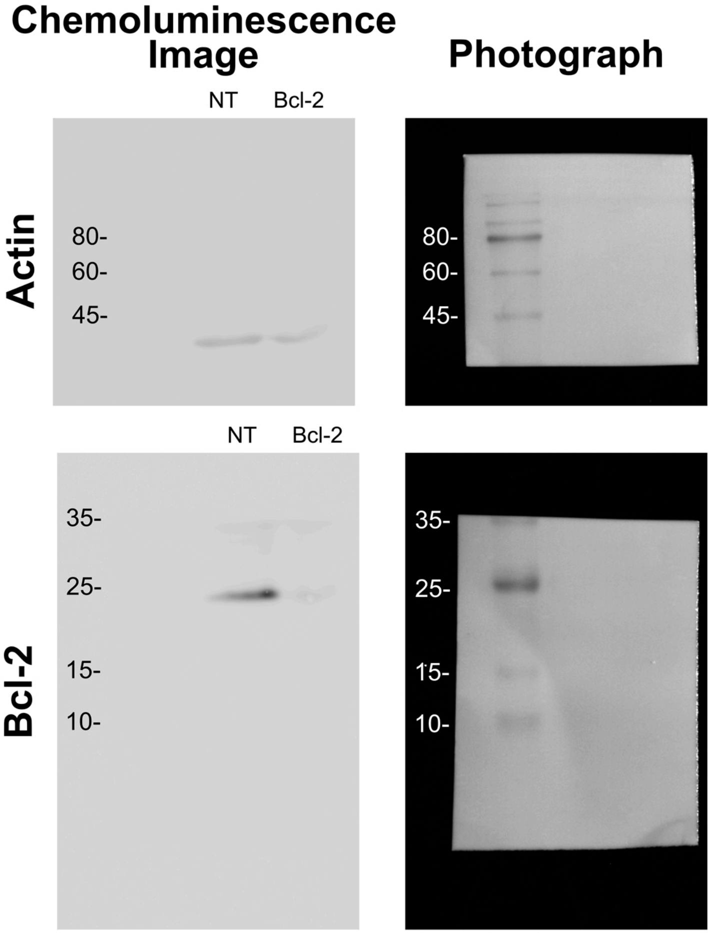

Supplementary Figure S17. Full length Western blot images of Bcl-2 silencing. Blue Plus IV Protein Marker (Trans) was used as a protein size marker. Actin was used as a loading control.

Full lenght Western blot images of Bcl-w silencing

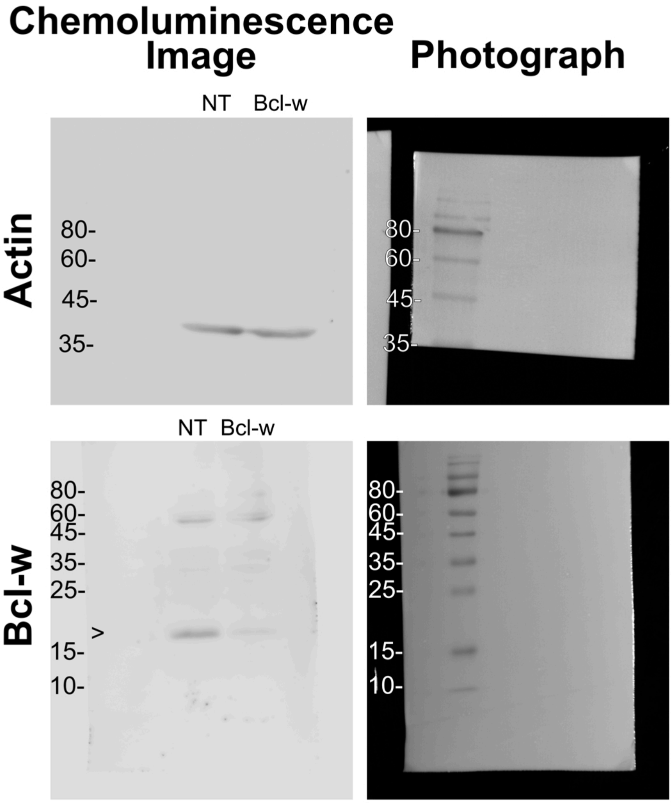

Supplementary Figure S18 Full length Western blot images of Bcl-w silencing. Blue Plus IV Protein Marker (Trans) was used as a protein size marker. Actin was used as a loading control.

Full lenght Western blot images of Bcl-xL silencing

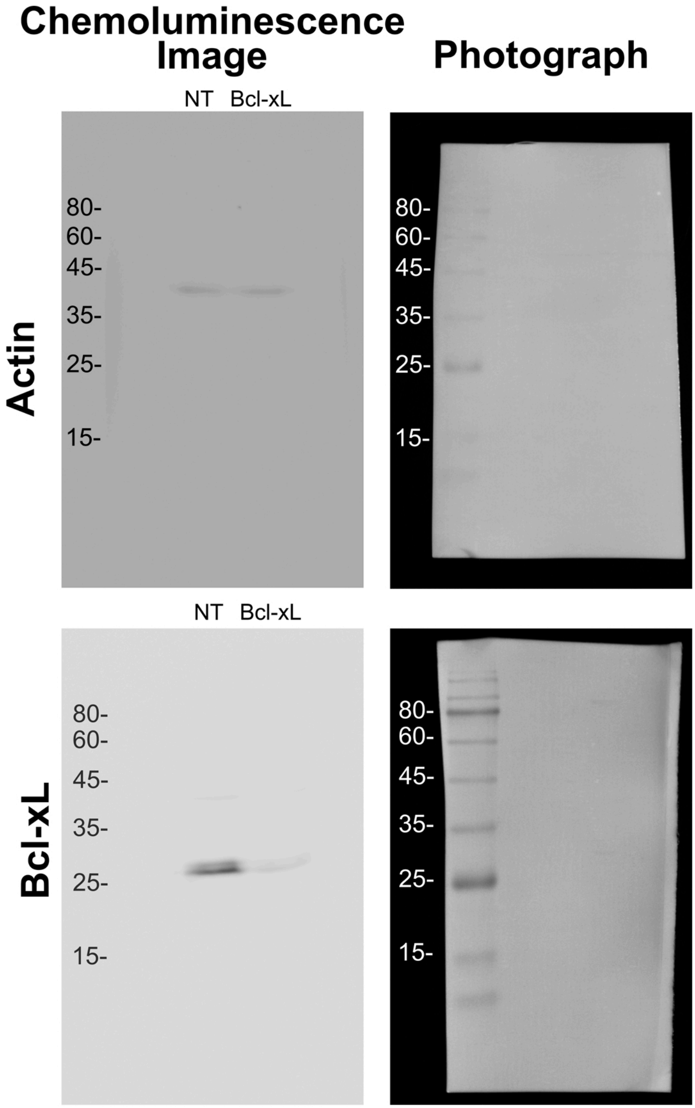

Supplementary Figure S19 Full length Western blot images of Bcl-xL silencing. Blue Plus IV Protein Marker (Trans) was used as a protein size marker. Actin was used as a loading control.

Full lenght Western blot images of Mcl-1 silencing

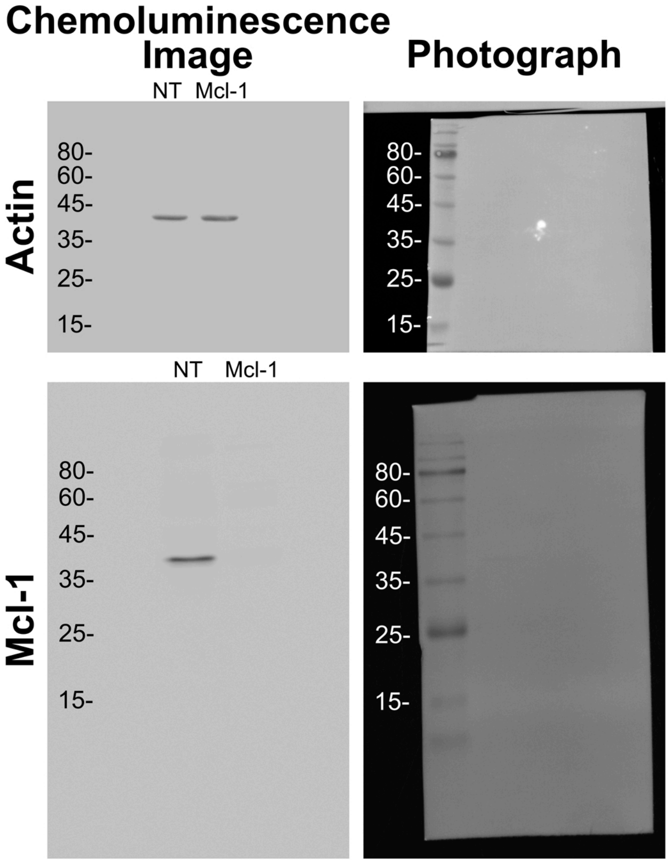

Supplementary Figure S20 Full length Western blot images of Mcl-1silencing. Blue Plus IV Protein Marker (Trans) was used as a protein size marker. Actin was used as a loading control.

Full lenght Western blot images of Noxa silencing

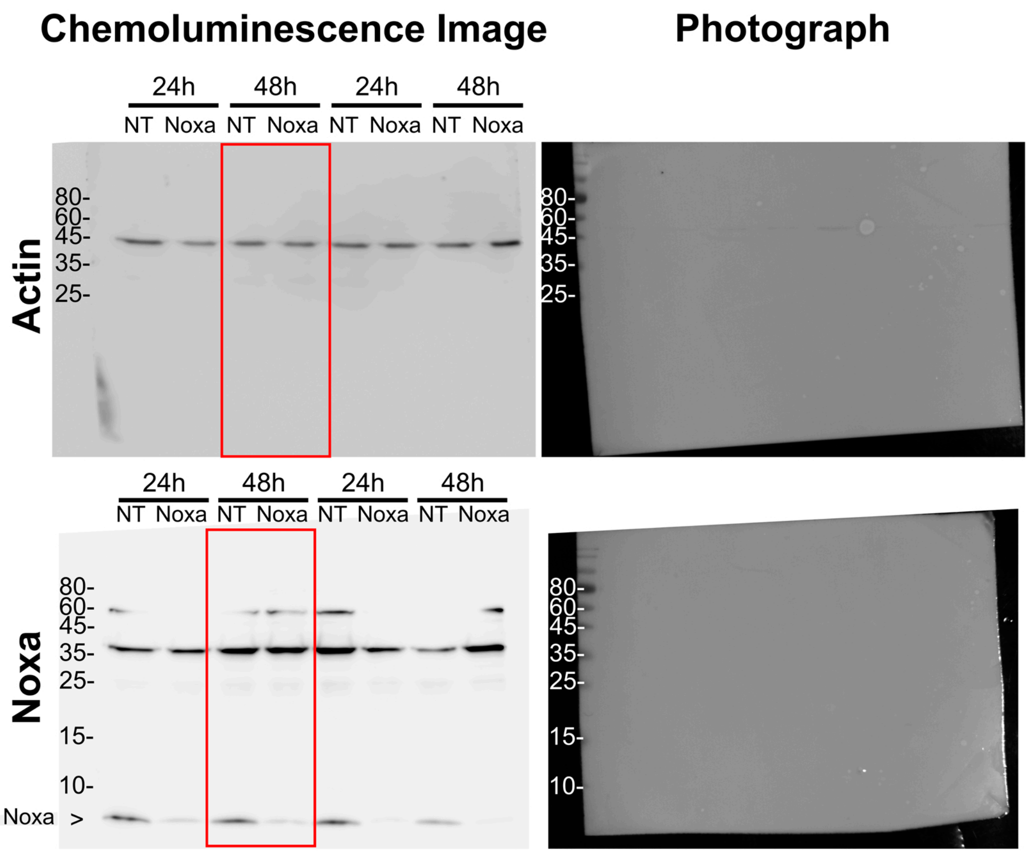

Supplementary Figure S21 Full length Western blot images of Noxa silencing. Blue Plus IV Protein Marker (Trans) was used as a protein size marker. Actin was used as a loading control. Relevant bands are marked by the red box.

Full lenght Western blot images of Noxa protein levels in treated G01 cell line

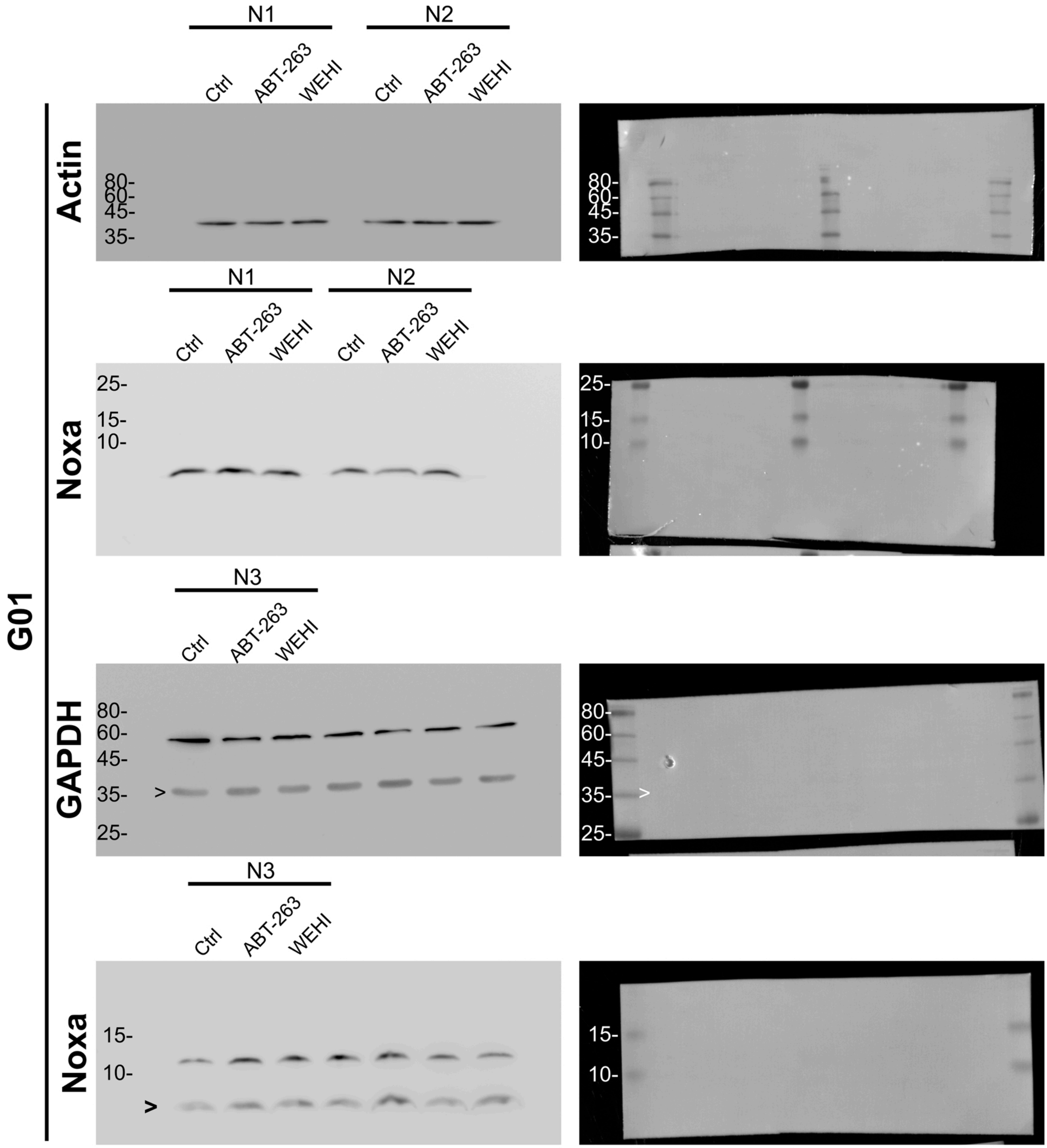

Supplementary Figure S22. Full length Western blot images of Noxa protein levels in response to treatment in G01. Blue Plus IV Protein Marker (Trans) was used as a protein size marker. Actin and GAPDH were used as loading controls. Relevant bands are indicated with an arrow.

Full lenght Western blot images of Noxa protein levels in treated G03 cell line

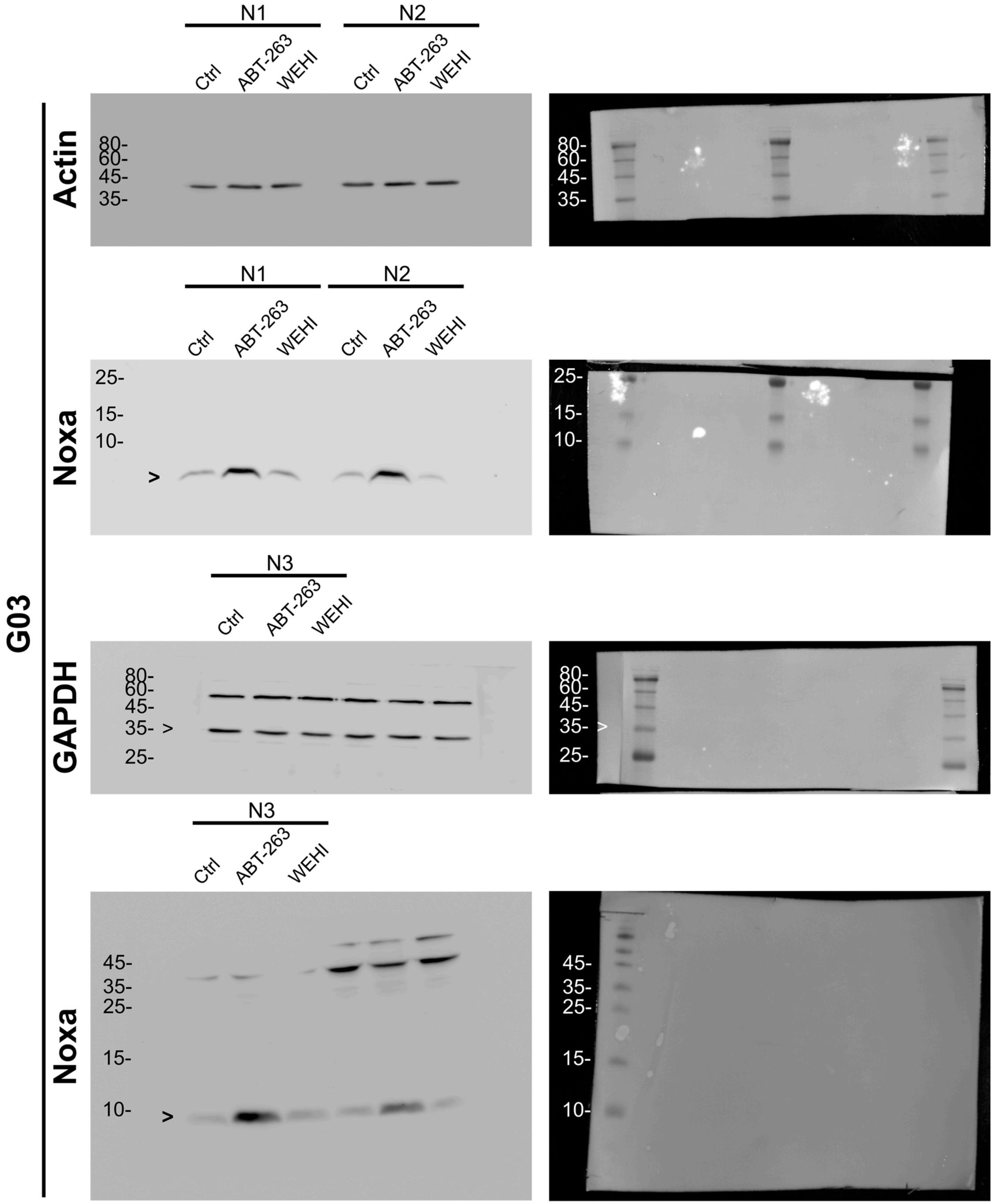

Supplementary Figure S23 Full length Western blot images of Noxa protein levels in response to treatment in G03. Blue Plus IV Protein Marker (Trans) was used as a protein size marker. Actin and GAPDH were used as loading controls. Relevant bands are indicatad by an arrow.

Supplementary Table S1

Cell line	Unmodified Mcl-1 expression (+ WEHI-539)			siRNA-silenced Mcl-1 expression (+ WEHI 539)		
	NT siRNA cell death (%)	Noxa siRNA cell death (%)	Δ Cell death (%)	NT siRNA cell death (%)	Noxa siRNA cell death (%)	Δ Cell death (%)
**G01**	30.9 ± 0.7	24.7 ± 1.0	**6.2 ± 0.6**	71.4 ± 9.0	49.6 ± 5.1	**21.7 ± 3.9**
**G02**	6.5 ± 1.5	6.6 ± 1.2	**-0.1 ± 0.8**	20.1 ± 2.2	14.1 ± 2.2	**6.0 ± 0.4**
**G03**	6.8 ± 0.4	4.6 ± 0.6	**2.2 ± 0.3**	36.0 ± 2.2	14.1 ± 2.0	**21.9 ± 1.2**
**G09**	7.2 ± 1.3	9.2 ± 0.6	**-2.0 ± 1.0**	60.5 ± 2.3	43.3 ± 2.9	**17.2 ± 4.2**

Supplementary Table S1. Cell death percentages depicted in figures 7a (unmodified Mcl-1 expression) and 8b (siRNA-silenced Mcl-1 expression). Values correspond to the mean ± S.D. of three independent experiments.
